# Stability of the cytosine methylome during post-testicular sperm maturation in mouse

**DOI:** 10.1371/journal.pgen.1009416

**Published:** 2021-03-04

**Authors:** Carolina Galan, Ryan W. Serra, Fengyun Sun, Vera D. Rinaldi, Colin C. Conine, Oliver J. Rando

**Affiliations:** Department of Biochemistry and Molecular Pharmacology, University of Massachusetts Medical School, Worcester, Massachusetts, United States of America; Walter and Eliza Hall Institute of Medical Research, AUSTRALIA

## Abstract

Beyond the haploid genome, mammalian sperm carry a payload of epigenetic information with the potential to modulate offspring phenotypes. Recent studies show that the small RNA repertoire of sperm is remodeled during post-testicular maturation in the epididymis. Epididymal maturation has also been linked to changes in the sperm methylome, suggesting that the epididymis might play a broader role in shaping the sperm epigenome. Here, we characterize the genome-wide methylation landscape in seven germ cell populations from throughout the male reproductive tract. We find very few changes in the cytosine methylation landscape between testicular germ cell populations and cauda epididymal sperm, demonstrating that the sperm methylome is stable throughout post-testicular maturation. Although our sequencing data suggested that caput epididymal sperm exhibit a highly unusual methylome, follow-up studies revealed that this resulted from contamination of caput sperm by extracellular DNA. Extracellular DNA formed web-like structures that ensnared sperm, and was present only in sperm samples obtained from the caput epididymis and vas deferens of virgin males. Curiously, contaminating extracellular DNA was associated with citrullinated histone H3, potentially resulting from a PAD-driven genome decondensation process. Taken together, our data emphasize the stability of cytosine methylation in mammalian sperm, and identify a surprising, albeit transient, period during which sperm are associated with extracellular DNA.

## Author summary

In mammals, after completing development in the testis, sperm continue to mature as they transit a long convoluted tube known as the epididymis. This process entails extensive molecular remodeling, with changes to the lipid composition of the sperm membrane, gain of key proteins, and a dramatic overhaul of sperm RNA contents. Here, to explore whether cytosine methylation patterns might also be edited during this process, we characterized cytosine methylation genome-wide at seven stages of sperm development in the testis and epididymis. Overall, we find remarkably stable methylation patterns throughout, supporting the classical view of mammalian cytosine methylation dynamics. In addition, while our data initially suggested a major methylation remodeling event in the caput epididymis and vas deferens, followup studies revealed that the aberrant methylation signature resulted from contamination by cell-free DNA. Curiously, this DNA was associated with citrullinated histones, suggesting that it might be generated by a cell death process akin to NETosis. While it will be interesting to determine whether epididymal cell-free DNA serves any biological function, the more immediate implication of our results is technical, as cell-free DNA could contaminate studies of the sperm epigenome, and so should be eliminated to improve the robustness of sperm methylation measurements.

## Introduction

In addition to contributing a haploid genome to the next generation, germ cells also deliver epigenetic information to progeny that can impact early development and later phenotypes. In mammals, this is best characterized in the context of genomic imprinting, a situation where genes exhibit monoallelic expression from either the maternal or paternal allele. In the classic examples of imprinted gene regulation, the heritable marking of imprinting control regions relies on the covalently-modified cytosine derivative 5-methylcytosine [1–4]. In mammals, cytosine methylation typically occurs in the context of CpG dinucleotides, and the genomic methylation landscape is copied every S phase by the maintenance methyltransferase DNMT1.

Cytosine methylation patterns in mammals undergo two major reprogramming events; the first during primordial germ cell development, and the second occurring upon fertilization. In the zygote, sperm methylation is rapidly erased shortly after fertilization, apparently via active demethylation, while oocyte methylation patterns are lost more slowly via passive demethylation (replication without maintenance methylation). However, a small number of genomic loci escape this demethylation process, including imprinting control regions and a subset of evolutionarily-young repeat elements [5,6]. The mechanisms responsible for protecting these loci from demethylation are still being uncovered, but recent studies implicate sequence-specific DNA binding proteins (ZFP57 and ZFP445) in maintenance of methylation levels at a subset of imprinting control regions [7,8].

Although the majority of the sperm methylation landscape is erased upon fertilization, the existence of escaper loci suggests the possibility that environmentally-regulated changes to the sperm methylome could play a role in modulating phenotypes in the next generation. Indeed, several studies have documented changes to sperm cytosine methylation in response to various diets or toxin exposures [9–13], raising the question of how the sperm methylome is regulated by environmental conditions.

Intriguingly, an early study on sperm methylation suggested the surprising possibility of a third cycle of methylation reprogramming, occurring during post-testicular sperm maturation in the epididymis [14]. Briefly, using methylation-sensitive restriction enzymes, the authors showed that methylation levels at two genomic loci appeared to change as sperm entered the proximal, or caput, epididymis. Given recent findings that another epigenetic information carrier in sperm, the small RNA payload, is extensively remodeled during epididymal transit [15–18], we envisioned the exciting possibility that the epididymis may play a broader role in control of the heritable sperm epigenome.

**Fig 1:**
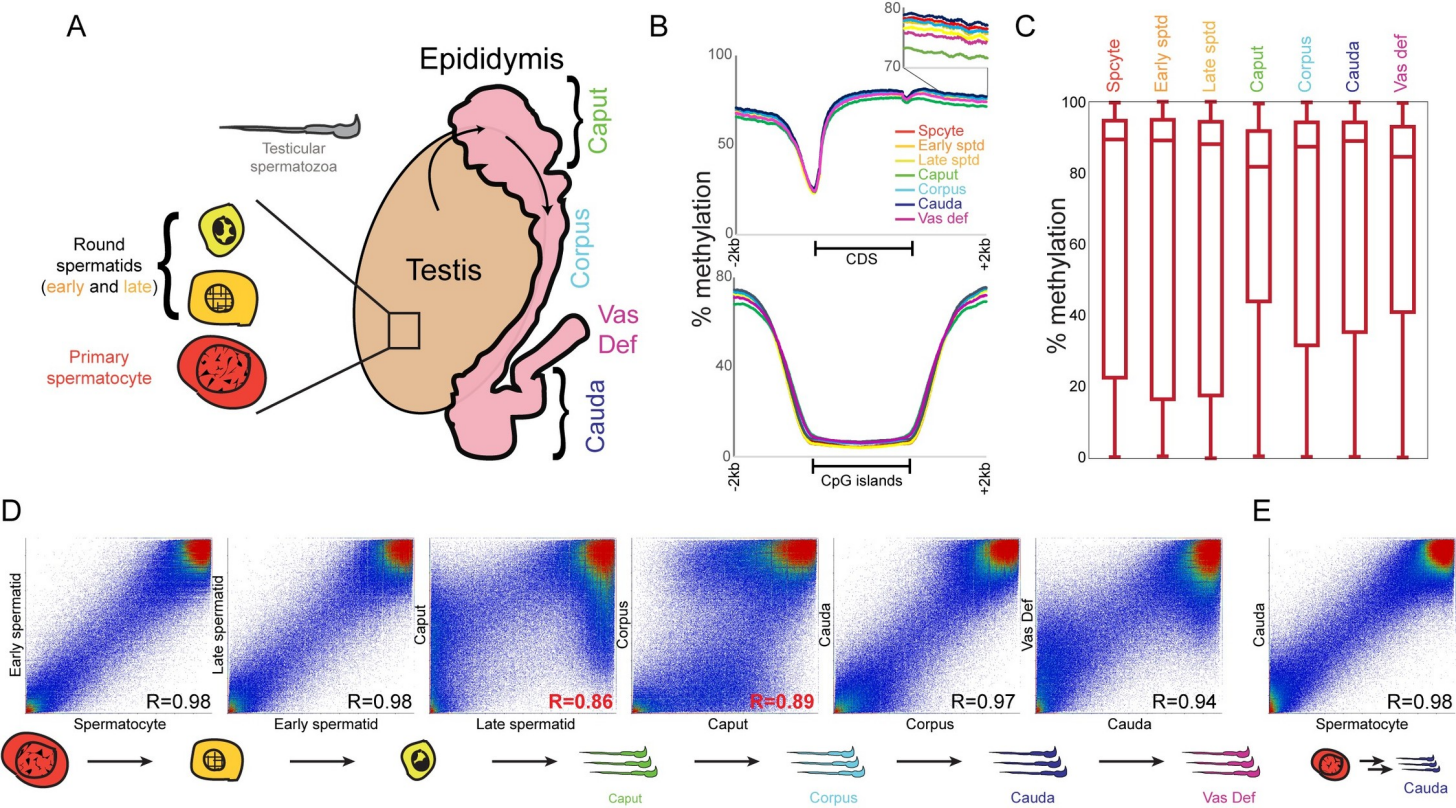
Whole genome cytosine methylation in seven germ cell populations. A) Schematic of seven germ cell populations analyzed in this study: primary spermatocytes, early and late round spermatids from testis, and spermatozoa from the caput, corpus, cauda epididymis and from the vas deferens. Shown in grey are mature testicular spermatozoa, which were not analyzed in our WGBS dataset. B) Typical features of the germline methylome were reproduced in all seven datasets. Top panel shows average methylation profile for metagenes normalized to the same length, along with 2 kb of sequence upstream and downstream of genes. Bottom panel shows methylation data surrounding all annotated CpG islands. C) Box plots show methylation levels for all 200 bp tiles across the genome. As expected, the majority of samples are overwhelmingly methylated, with caput sperm and vas deferens sperm exhibiting modest hypomethylation relative to the other five samples. D) Scatterplots comparing methylation levels for consecutive sperm developmental stages. In each case, scatterplot shows methylation levels averaged for all 200 bp tiles (with at least 10 methylation-informative reads) across the genome. Overall, all samples exhibit robustly correlated methylation landscapes with one another, with the caput and vas deferens samples representing outliers with hundreds of hypo- and hyper-methylated tiles compared to other sperm samples. See also **S2 Fig** for all pairwise comparisons. E) Scatterplot comparing methylation levels in primary spermatocytes and cauda sperm, showing that the genomic loci that exhibit changes in methylation in caput sperm return to their original methylation levels later in the epididymis.

Motivated by the Ariel *et al* study, we therefore revisited cytosine methylation dynamics during post-testicular maturation, using the gold standard whole genome bisulfite sequencing (WGBS) to characterize the methylation landscape genome-wide, at single-nucleotide resolution, in seven germ cell populations from primary spermatocytes to vas deferens spermatozoa (**Fig 1A**). We found remarkably consistent methylation profiles in five of the seven germ cell populations, with strong correlations between the various testicular germ cell populations and corpus and cauda epididymal sperm. This general persistence of methylation patterns was interrupted by caput epididymal sperm, which exhibited modest global hypomethylation accompanied by hypermethylation of germline-associated CpG islands. We ultimately identified cell-free DNA, presumably derived from somatic cells of the epididymis, as the cause of the unusual “caput sperm” methylome–most definitively, we show that treating caput or vas deferens sperm with DNase I prior to sperm lysis restored the aberrant sperm methylation landscape to the same pattern seen in all other sperm populations from testicular through cauda epididymal sperm. Cell-free DNA was associated with the citrullinated histone H3 (citH3) that is characteristic of arginine deimination by peptidylarginine deiminase (PAD) enzymes, and, curiously, was detected only in virgin males. Taken together, our data support a static view of methylation patterns stably persisting throughout post-testicular sperm development, and reveal an intriguing but transient stage of programmed cell-free DNA production in the male reproductive tract.

## Results

### Genome-wide analysis of the sperm cytosine methylation landscape

We set out to build on prior low-throughput studies documenting differences in cytosine methylation between testicular sperm and sperm obtained from various regions of the epididymis [14]. To this end, we collected seven germ cell populations from 10–12 week old FVB males: primary spermatocytes, two populations of round spermatids, and spermatozoa obtained from the caput, corpus, and cauda epididymis, and from the vas deferens (**Fig 1A**). For each population we collected samples from seven different males, isolated and pooled genomic DNA, and prepared libraries for whole genome bisulfite sequencing (WGBS), obtaining an average of ~300 million reads per pool (**S1 Table**). The resulting data recapitulate well-described features of the sperm methylome [19–22], such as a high overall level of methylation punctuated by hypomethylated CpG islands (**Figs 1B, 1C** and **S1**), supporting the quality of our dataset.

### A transient methylation signature in the caput epididymis interrupts otherwise stable methylation throughout sperm maturation

We next turned to the question of how the sperm methylome changes over the course of epididymal transit. Comparing all seven populations, we noted similar overall methylation profiles, with global methylation interrupted by hypomethylated CpG islands in all seven samples (**Fig 1B and 1C**). Intriguingly, we found somewhat lower global methylation in caput sperm and, to a lesser extent, in sperm from the vas deferens. Examination of metagenes revealed that these two sperm populations deviated from the characteristic methylation profile observed in the other five samples, with lower methylation levels across coding regions, along with subtly increased methylation at promoters (**Fig 1B** and see below).

To more systematically search for methylation changes between germ cell populations, we averaged methylation levels over 200 bp regions tiled across the genome and compared methylation levels in these tiles between all pairs of samples in our dataset (**Figs 1D** and **S2**). Overall, we found that five germ cell populations exhibited nearly-identical methylation landscapes, with strong correlations between the methylation datasets for the three testicular samples, and for corpus and cauda sperm. Importantly, this means that the differences in methylation between round spermatids and caput sperm are reversed in corpus sperm, rather than these methylation changes being part of an ongoing process of progressive methylation maturation in the epididymis. In other words, the nearly-identical methylation profiles for testicular spermatocytes/spermatids and cauda sperm (**Fig 1E**) indicates that the sperm methylome is essentially unchanged by the process of epididymal maturation, and strongly argues against a major role for the epididymis in modulating sperm methylation.

Below, we explore the unusual methylome of caput and vas deferens sperm. Given the similar behavior of vas deferens and caput sperm populations, we primarily focus in follow-up experiments on the more dramatic methylation changes observed in caput sperm, but we ultimately show that the aberrant methylation signatures of caput and vas deferens sperm result from similar processes.

### Widespread derangements in CpG island methylation in caput epididymal sperm

In contrast to the nearly-identical methylation profiles obtained from testicular and corpus/cauda sperm, comparisons between caput sperm and any of these samples revealed large-scale changes in methylation across hundreds of 200 bp tiles (**Figs 1D** and **S2**). Overall, as noted above, caput sperm were slightly less methylated than these other sperm samples–examination of hypomethylated loci in caput sperm revealed diffuse hypomethylation over a wide range of both coding and intergenic genomic loci (**S2 Table**). This signature was also detectable in metagene averages in the highly-methylated regions distant from CpG islands, with caput sperm exhibiting a small global deficit at these loci (**Fig 1B**, insets).

**Fig 2:**
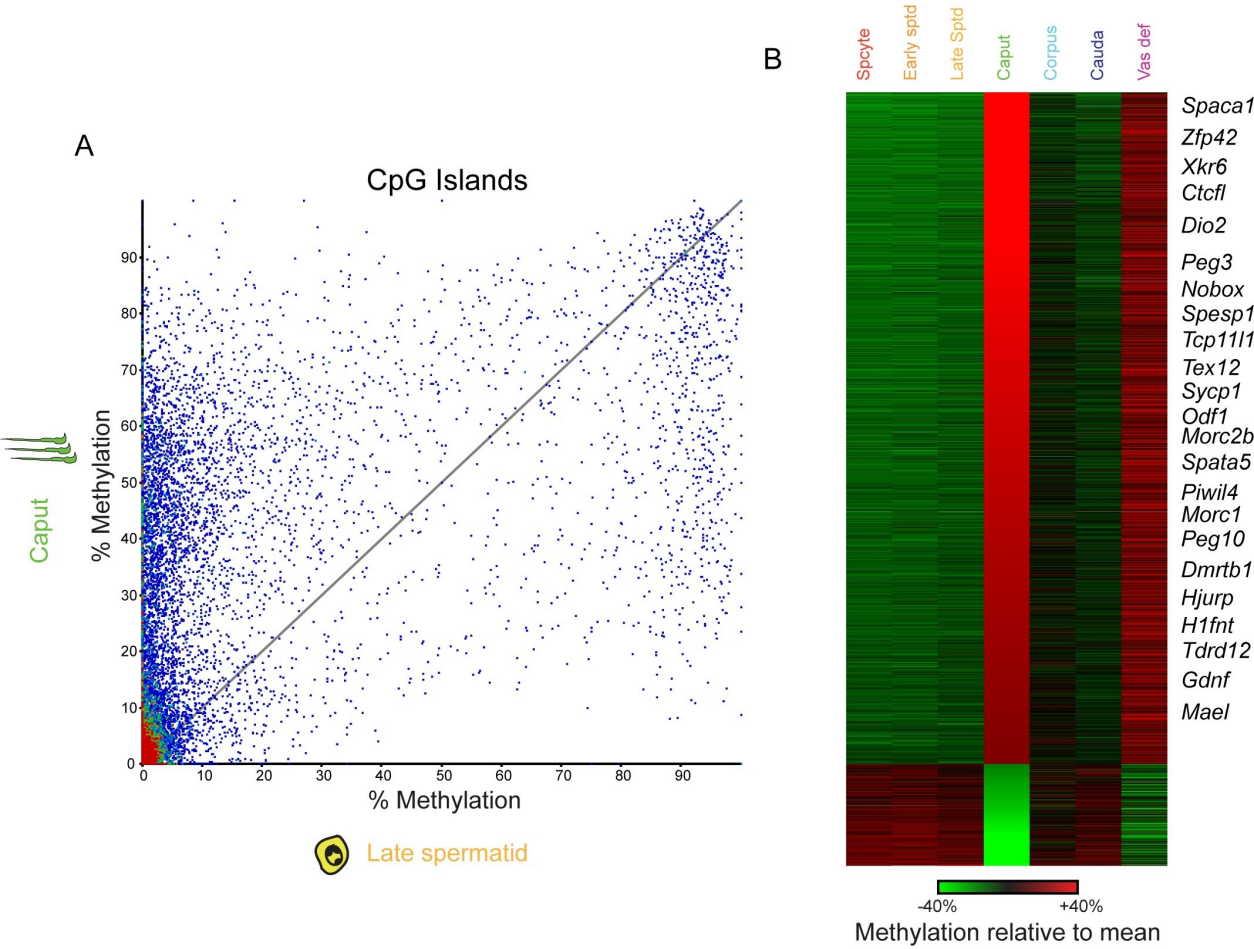
CpG island hypermethylation in caput and vas deferens sperm. A) Scatterplot showing average methylation levels across all annotated CpG islands, comparing late round spermatids and caput epididymal sperm. Although the majority of CpG islands are still hypomethylated in both samples (lower left corner), caput sperm are characterized by hundreds of abnormally hypermethylated CpG islands (dots above the x = y diagonal). B) Heatmap for CpG islands that are more than 20% hypo- or hyper-methylated in caput sperm relative to other samples. Each row represents the methylation values of a single CpG island, normalized relative to the average methylation % across all seven samples. Islands are sorted from hyper-methylated in caput sperm to hypo-methylated. As caput-hypermethylated CpG islands are enriched for those located near genes with known reproductive functions (**S3 Fig**), notable genes are annotated along the right of the heatmap. We note that the majority of caput-*hypo*methylated CpG islands (bottom of the heatmap) are located within transcribed regions, and may therefore reflect the modest general hypomethylation observed over transcribed regions (**Fig 1B and 1C**).

In addition to the widespread hypomethylation of the caput sperm genome, a large group of hypermethylated tiles was readily apparent in these scatterplots. Examination of these tiles revealed that they are largely associated with CpG islands. To visualize this, we calculated the average methylation across all annotated CpG islands; **Fig 2A** shows a scatterplot comparing CpG island methylation in round spermatids and caput sperm–nearly-identical results were obtained in comparisons between caput sperm and other testicular populations, or corpus/cauda epididymal sperm (**S3 Table**). Together, these analyses reveal extensive methylation changes at regulatory elements in caput sperm, primarily reflecting hypermethylation of CpG islands in this sample (dots above and to the left of x = y) with a relatively small number of hypomethylated islands in caput sperm. This is further illustrated in **Fig 2B**, which documents methylation dynamics for differentially-methylated CpG islands across all seven germ cell populations, highlighting the dramatic changes in CpG island methylation in caput sperm (and, to a lesser extent, in vas deferens sperm). Again, we note that regulatory elements that exhibit aberrant methylation in caput sperm universally return to the spermatocyte/spermatid methylation patterns in the corpus and cauda samples. In other words, whatever methylation changes apparently occur in caput sperm are soon reversed in later sections of the epididymis (with the exception of the vas deferens, discussed later).

What biological pathways are affected by epididymal methylation dynamics? To address this question, we sought gene ontology categories enriched in genes associated with caput sperm hypo- and hyper-methylated CpG islands (**S3 Fig**). Hypomethylated islands in caput sperm are significantly enriched for a handful of processes related to neuronal function (eg, neuronal cell body). However, these enrichments appear to be primarily driven by the fact that caput-hypomethylated CpG islands are found within transcribed regions–the hypomethylation at these loci presumably reflects the more general hypomethylation characteristic of caput sperm (**Fig 1B and 1C**). More intriguingly, *hyper*methylated islands are enriched for a variety of annotations associated with meiosis- and sperm-specific functions, including piRNA biogenesis, ion signaling, and reproductive process.

**Fig 3:**
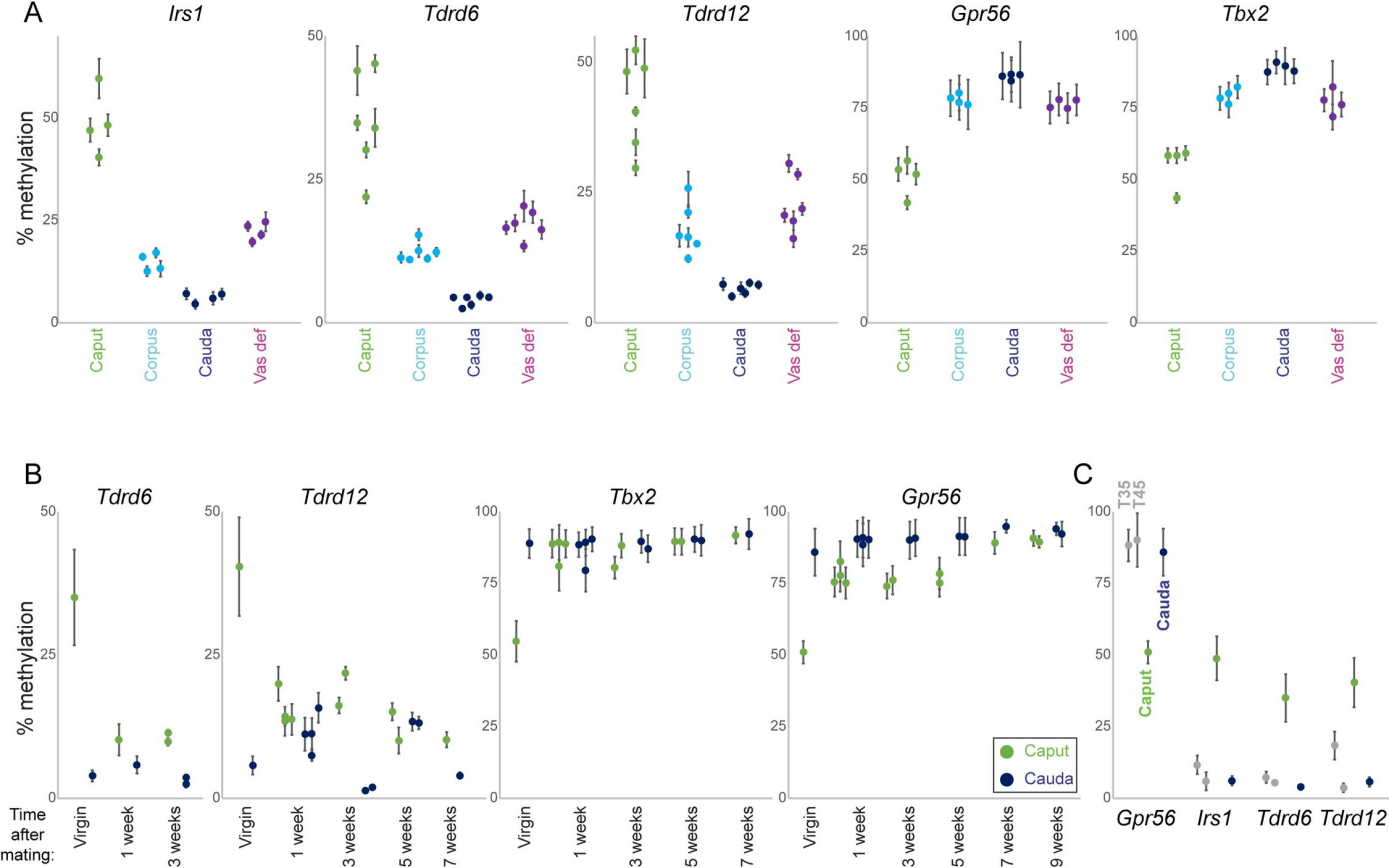
The unusual methylation program in caput sperm is lost after mating. A) Pyrosequencing validation of WGBS data. Pyrosequencing data are shown for five loci which exhibited caput-specific methylation levels in our WGBS dataset (**S4A Fig**). For each locus, pyrosequencing data are shown for 4–6 samples each of caput, corpus, and cauda epididymal sperm, as well as vas deferens sperm, as indicated. Animals were 10–14 weeks of age at time of collection. Error bars show standard deviation across multiple CpGs assayed at each locus. In all cases the pyrosequencing data recapitulate the differences between sperm populations identified in our WGBS dataset, and differences between caput and cauda methylation levels are highly statistically-significant (t test p values ranging from 1.2e-4 for *Tbx2* to 2.0e-6 for *Tdrd12*) for all five loci. B) The caput-specific methylation profile is specific to virgin males. Pyrosequencing data shown as in panel (A), for caput and cauda sperm obtained from virgin males or males sacrificed at varying times after confirmed mating. C) Testicular sperm obtained from prepubertal males (thus enriched with the first wave of spermatogenesis) exhibit similar methylation to cauda, not caput, sperm from mature animals. For each target locus, data are shown for testicular sperm obtained from 35 day or 45 day old males, as well as caput and cauda epididymal sperm obtained from 10–14 week old animals.

To validate our genome-wide dataset, and to develop a set of cost-effective targets for follow-up mechanistic studies, we analyzed methylation levels at a number of target loci (**S4 Fig**) by pyrosequencing of bisulfite-converted DNA. As shown in **Fig 3A**, methylation differences between caput and cauda were robustly reproducible in many additional, independent pairs of sperm samples. Moreover, the caput methylome proved to be quite stable over an animal’s lifespan–methylation at two target loci assayed remained abnormal in caput sperm obtained from ten month old animals (**S5 Fig**).

These data validate our overall dataset, and we focus on these target loci in the mechanistic follow-up studies described below. Importantly, although we considered the possibility that a signature of hypermethylation of germline regulatory elements might reflect contamination by somatic cells, we ensured that the methylation differences between caput and cauda sperm were robust to several sperm purification protocols including one based on detergent washing of caput epididymis luminal contents, and an alternative Percoll-based isolation of caput sperm in the absence of detergent treatment (**Methods**). For all samples we routinely determined, based on the characteristic hook-shaped morphology of the murine sperm head, that our caput sperm preps were >99% free of somatic cell contamination.

### The caput sperm methylome is stable in multiple buffer conditions

We next sought to identify the mechanistic basis for the widespread changes in methylation observed in caput sperm, to explore the molecular basis for what would represent a relatively rare case of fully replication-independent cytosine demethylation. One of the signature functions of the mammalian epididymis is to provide a variety of highly distinctive luminal microenvironments that serve a multitude of functions in supporting sperm maturation and preventing premature activation [23,24]. It is well known that the ionic composition differs significantly between different luminal compartments, as for example pH and calcium levels vary dramatically between caput and cauda, and the extensive gene expression differences between different segments [25,26] imply that many other metabolites will also differ in concentration throughout the epididymis.

We therefore set out to test the hypothesis that sperm methylation dynamics result from “pre-loaded” genome-associated DNA modification enzymes (DNMTs for methylation, TET enzymes for demethylation) whose ongoing activity could be inhibited or activated by changes in either substrate levels (SAM, alpha-ketoglutarate, iron oxidation status, etc.) or buffer conditions (DNMT3a activity is highly pH-dependent [27]). To test this, we attempted to recapitulate the caput to cauda methylation changes by incubating purified caput sperm in various buffer conditions meant to mimic the cauda epididymal lumenal environment (**S6 Fig**). However, none of the buffer conditions tested were able to substantially influence the caput sperm methylome.

### The caput sperm methylome is lost following mating

During the course of these studies, we found one animal in which the methylation level at our target genes was nearly identical for caput and cauda sperm. Further investigation revealed that a female had accidentally been weaned into an otherwise all-male cage, suggesting that mating might influence the methylation dynamics described above. Indeed, direct testing of this hypothesis confirmed that methylation of our target genes was nearly identical in caput and cauda sperm obtained from animals who had successfully sired offspring (**Fig 3B**). This was due to the caput methylation profile shifting to match that of cauda sperm; cauda sperm methylation was totally unaffected by the male’s mating status.

We considered and rejected two hypotheses regarding the change in methylation status of caput sperm in mated animals: 1) that conversion of the testicular sperm methylome to the unusual caput state might require extended incubation of newly-arrived sperm in the caput luminal environment, and 2) that the virgin caput methylation profile reflects sperm originating from the unusual first meiotic wave of spermatogenesis and somehow being captured in the caput epididymis. The first hypothesis was rejected based on the finding that the cauda-like methylation program in caput sperm from mated animals was stable for at least nine weeks following mating (**Fig 3B**). We next considered the possibility that caput sperm obtained from virgin animals represent an unusual first wave of spermatogenesis [28] characterized by an atypical methylation program. This hypothesis would require an unlikely process–in which first wave sperm are slowed or arrested in the proximal epididymis, allowing subsequent waves of sperm to pass into the corpus and cauda epididymis–but we could not rule out the hypothesis a priori. However, enriching first wave sperm by isolation of testicular spermatozoa from 35 day-old animals revealed the same methylation levels at our validation loci as those observed for testicular and cauda sperm from older animals (**Fig 3C**), refuting the hypothesis that first wave sperm carry an unusual methylome.

### Contamination of caput epididymal sperm by cell-free DNA

**Fig 4:**
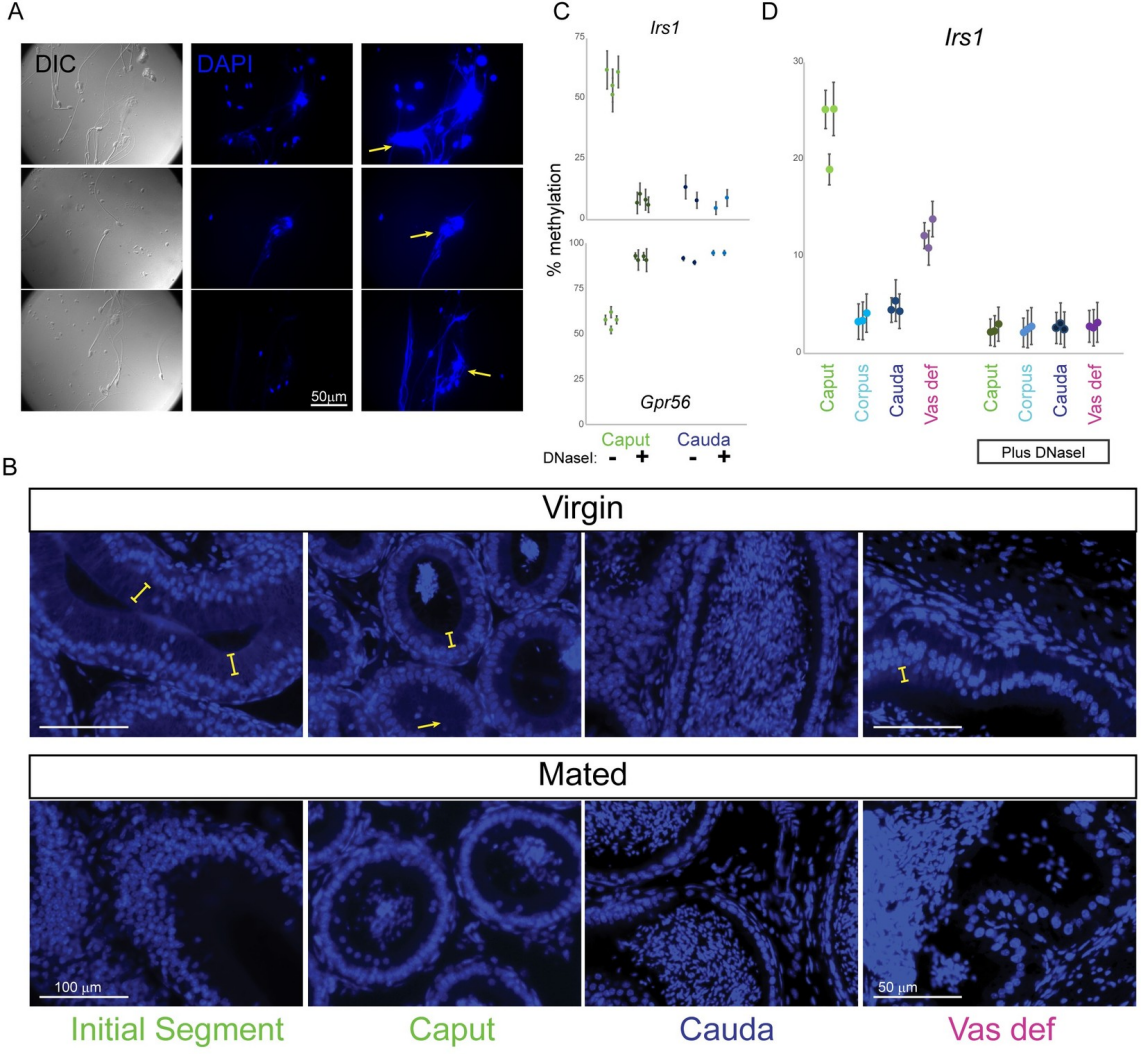
Cell-free DNA in the caput epididymis of virgin males is responsible for the caput methylome. A) DAPI staining of caput sperm samples (see **S7 Fig** for cauda sperm samples). For each sample, left panel shows DIC to highlight sperm locations, middle and right panels show DAPI staining, with far right panel showing longer exposures. Yellow arrows indicate examples of web-like cell-free DNA in caput sperm samples. B) In situ DAPI staining of the epididymis. Images show DAPI-stained histology sections of the initial segment, caput epididymis, cauda epididymis, and vas deferens of either virgin or mated males, as indicated. Yellow brackets indicate examples of DAPI-positive rim associated with the apical regions of the epithelium of initial segment or caput samples from virgin males. Arrow shows a lumen nearly completely filled with this material. See **S8 Fig** for additional in situ staining images. C) Cell-free DNA is responsible for the abnormal caput methylome. Pyrosequencing data are shown for caput and cauda sperm samples, either mock treated or pre-treated with DNase I prior to genomic DNA purification. At both loci, differences between untreated caput and cauda sperm are again highly significant (p < 1e-5), consistent with **Fig 3A**, while there are no significant differences between either DNase-treated caput sperm and cauda sperm, or between treated and untreated cauda sperm (p > 0.05 in all cases). D) DNase-sensitive aberrant methylation is consistently observed in multiple strain backgrounds. Pyrosequencing data for *Irs1* are shown for the indicated sperm samples obtained from three 10–14 week old C57Bl6/J males. Left samples show methylation levels for genomic DNA isolated from washed sperm pellets, while right samples show data for genomic DNA isolated following DNase treatment of sperm pellets. As observed for the FVB strain background used throughout the rest of the manuscript, untreated caput and vas deferens sperm preps from C57 animals also exhibit aberrant methylation which is completely corrected following DNase I treatment. See also **S9 Fig**.

What then could account for the unique methylation program observed in caput sperm from virginal males? Careful inspection of our genomic DNA preparations revealed that although testicular and cauda sperm samples were characterized by uniformly high molecular weight genomic DNA, there was a dim additional “cloud” of lower molecular weight DNA in the caput sperm preps. This suggested the possibility that caput sperm might be contaminated by cell-free DNA. Indeed, DAPI staining of caput sperm preparations revealed not only the expected hook-shaped sperm heads, but at higher exposure times we noted the presence of web-like DNA structures, often ensnaring multiple sperm (**Fig 4A**). These webs were not detected in cauda epididymal sperm preparations (**S7 Fig**). To visualize this extracellular DNA in situ, we stained histological sections of several epididymal regions with DAPI. Intriguingly, in both caput sections and in the vas deferens we find a DAPI-staining rim associated with the apical region of the epithelium (**Figs 4B** and **S8**). DAPI-positive rims were not observed in the cauda epididymis, and were diminished or lost from the caput epididymis and vas deferens following mating (**Fig 4B**, bottom panels). We cannot be certain that these DAPI rims are the source of the free DNA that contaminates caput sperm preps–the DAPI rims appear to coincide with the cell bodies of the epididymal epithelium (**S8 Fig**)–but it is notable that these rims were only observed in the same samples (caput but not cauda epididymis, virgin animals but not mated animals) that suffer from cell-free DNA contamination.

To definitively test whether extracellular DNA is responsible for the methylation profile of virginal caput sperm, we treated various sperm samples with DNase I to eliminate any extracellular DNA prior to extraction of genomic DNA for pyrosequencing analysis. Remarkably, DNase I treatment completely restored the caput sperm methylome to the methylation levels observed in testicular or cauda epididymal sperm (**Figs 4C and S9A**), demonstrating that contaminating cell-free DNA present in the caput epididymis is responsible for the unusual caput methylome observed in virgins. To ensure that this finding was not idiosyncratic to the FVB/NJ strain background used throughout this study, we also carried out pyrosequencing in epididymal and vas deferens sperm samples obtained from C57Bl6/J males (**Figs 4D and S9B**), again confirming 1) that caput and vas deferens sperm exhibited aberrant methylation at our target genes in this strain background, and 2) that this aberrant methylation profile was eliminated by DNase treatment of sperm preparations prior to sperm lysis and genomic DNA extraction.

**Fig 5:**
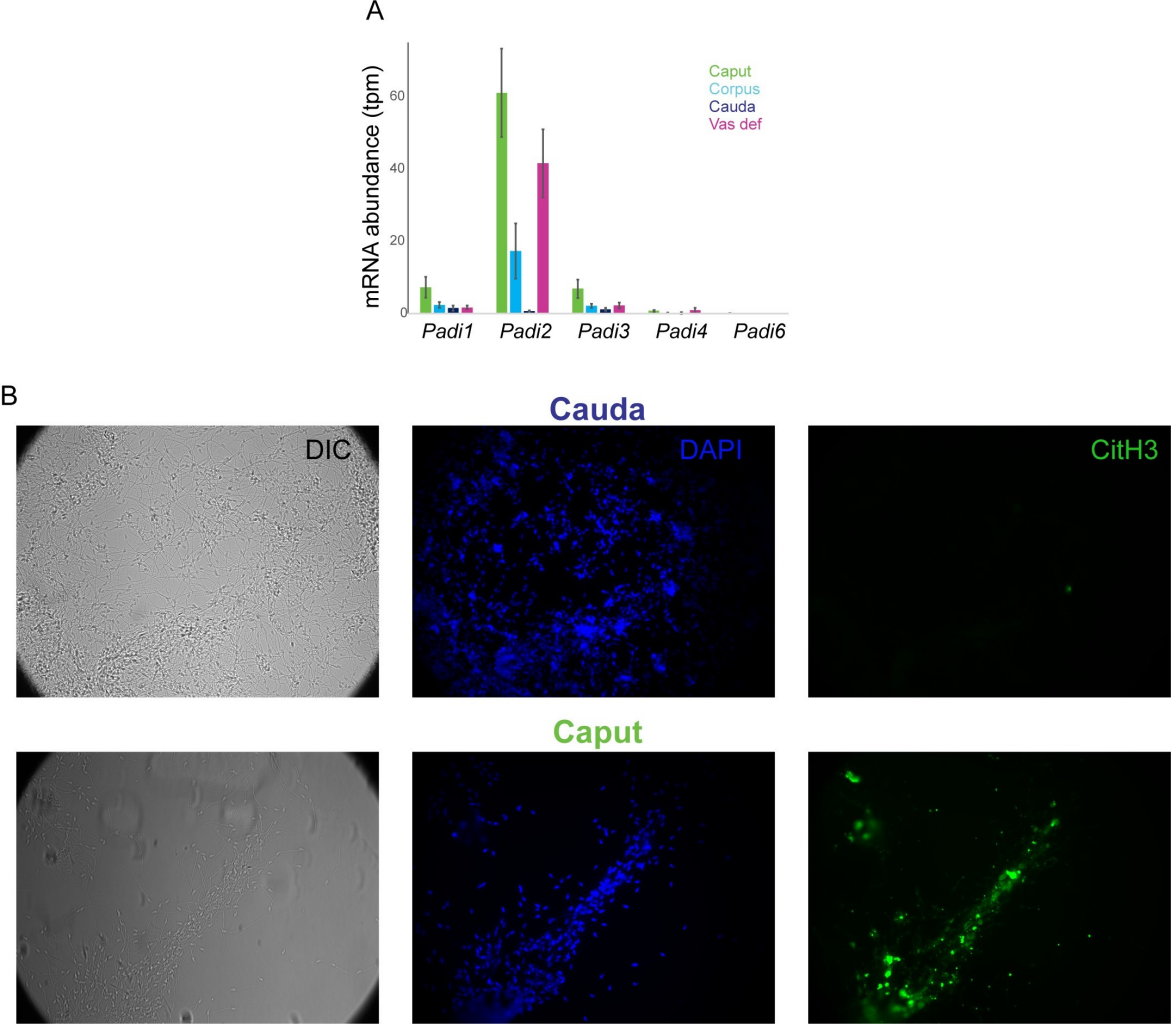
Cell-free DNA is produced via a NETosis-like process. A) Expression of PAD-encoding genes in the murine epididymis and vas deferens. RNA-Seq data are from [32]. B) Caput epididymal cell-free DNA is associated with citrullinated histone H3. Caput and cauda sperm samples were stained for DAPI (blue) and for citH3 (green), as indicated.

Based on its impact on caput sperm methylation, it seems likely that the cell-free DNA originates from somatic cells rather than germ cells (see **Discussion**). We note that the cell-free DNA observed in our sperm preparations is reminiscent of extracellular DNA released by neutrophils, known as Neutrophil Extracellular Traps, or NETs [29–31]. The process of NETosis is associated with increased peptidyl arginine deiminase (PAD) activity, resulting in arginine deimination on histones and other proteins, leaving behind citrulline and leading to massive chromatin decondensation. Examination of RNA-Seq data from throughout the epididymis [32] confirmed expression of *Padi2* in this tissue, largely confined to principal cells of the caput epididymis and vas deferens (**Fig 5A**). Moreover, consistent with the caput cell-free DNA being produced downstream of PAD-dependent deimination, we found robust citH3 staining of our caput sperm preparations, with negligible signal in cauda sperm preps (**Fig 5B**).

Taken together, our data reveal that extracellular DNA, potentially produced via a PAD-associated process, contaminates sperm obtained the caput epididymis and vas deferens of young males, and that this cell-free DNA is depleted following mating.

## Discussion

Here, we characterized the cytosine methylation landscape during late stages of spermatogenesis, and throughout post-testicular sperm maturation in the epididymis. Most importantly, these data reveal that the sperm methylome is quite stable during the process of post-testicular maturation. This is clearest in **Fig 1E**, which shows the robust correlation between the methylation patterns in primary spermatocytes and in mature cauda epididymal sperm. Our data therefore do not support a model in which sperm methylation is extensively remodeled during epididymal transit. Although we cannot rule out subtle methylation changes occurring in epididymal sperm in response to environmental stressors, the absence of changes in this dataset strongly supports the classic view of germline cytosine methylation patterns as largely static in the absence of replication-coupled remodeling events.

Our study also revealed a surprising feature of the male reproductive tract, in which cell-free DNA is present in the caput epididymis and the vas deferens of virgin males. This DNA clearly derives from somatic cells rather than dying sperm, since the methylation profile of virgin caput sperm departs from the characteristic germline methylome to incorporate aspects more typical of somatic cells. This is apparent in the hypermethylation of key germline regulatory elements, which are unmethylated and active during spermatogenesis but methylated and repressed in most somatic tissues. This is also clear at imprinting control regions, which are either completely methylated or unmethylated in the sperm genome, but are 50% methylated in somatic cells–the contaminated caput methylome exhibits a shift towards 50% methylation at many imprinting control regions (**S4C Fig**). Based on the methylation levels at imprinted regulatory elements, as well as changes in DNA yield following DNase I treatment of caput sperm prior to gDNA isolation, we roughly estimate that in our hands caput sperm genomic DNA is contaminated by ~20% somatic cells. Of course, this level of contamination by intact cells would readily be detectable by inspection of our caput sperm preps, emphasizing the fact that this contamination arises from cell-free DNA rather than intact somatic cells. That said, it remains plausible that the contaminating DNA comes from cells that lyse during dissection and sperm preparation, although it would be unusual for such DNA to be associated with citrullinated histones.

It is unclear which cells are responsible for production of cell-free DNA. Extracellular DNA traps are most famously produced by neutrophils during a process known as NETosis, but they have been reported in multiple inflammatory cell types [33,34]. In this regard it is intriguing that sperm have been shown to induce NETosis when coincubated with leukocytes [35]. Nonetheless, we favor the hypothesis that the extracellular DNA in the caput epididymis originates in principal cells of the epididymal epithelium. This is based on 1) the rarity of neutrophils in the epididymis [32]; 2) the expression of *Padi2*, rather than *Padi4* which is more typically associated with NETosis, in our RNA-Seq dataset (**Fig 5A**); 3) our inability to detect substantial staining of the neutrophil marker myeloperoxidase in the caput cell-free DNA (not shown); and 4) the expression of *Padi2* specifically in principal cells in our single-cell atlas of the epididymis [32]. Although in principle the identity of the cell of origin for this DNA could leave a signature in our methylation dataset, examination of methylation at several immune cell marker genes proved inconclusive.

Whatever the cell of origin of the extracellular DNA here, it seems likely that it is produced via a PAD-dependent genome decondensation event analogous to the one that drives the production of Neutrophil Extracellular DNA Traps, or NETs [30]. This hypothesis is motivated by the expression of *Padi2* specifically in the two regions exhibiting cell-free DNA, and by the confirmed presence of abundant citH3 staining in caput sperm preps (**Fig 5**). This raises the question of what purpose extracellular DNA in the epididymal lumen might potentially serve. Extracellular DNA plays a key role in defense against pathogens in several contexts–NETs in the mammalian bloodstream entangle pathogens and aid in their engulfment by antigen-presenting cells [30], while extracellular DNA in pea (*P*. *sativum*) roots was shown to protect against fungal infections [36]. While a similar role could be imagined for extracellular DNA in the epididymis helping to prevent ascending infections, it is notable that this material is present only in virgins but lost after mating (**Fig 3B**). As infections would presumably be more likely in sexually-active animals than in virgins, we therefore disfavor this hypothesis, although it is plausible that the system serves to ensure the reproductive machinery is protected until a first mating is achieved. Alternatively, we speculate that the DNA webs observed here could ensnare sperm rather than pathogens, potentially playing a role in removal of early rounds of defective sperm, or masking sperm from inducing an immune response. Of course, the simplest hypothesis would be that the extracellular DNA documented here is a vestige of some earlier developmental process–death of epithelial cells during epididymal morphogenesis, for instance–and serves no biological function. That said, the use of PAD-dependent cell death would be unusual for typical programmed cell death in development.

Although the function, if any, served by extracellular DNA in the epididymis is unclear, the more pressing implication of our findings is that they raise two important technical considerations for reproductive epigenetics studies. First, one important finding here is that at least some aspects of sperm populations are different between virgins and mated males, raising the question of whether other features of the epididymal sperm epigenome–often assayed in virgin males–are stable throughout reproductive life. For example, we and others have previously documented substantial differences between the small RNAs carried by caput and cauda epididymal sperm, with a variety of genomically-clustered microRNAs, and a subset of tRNA fragments such as tRF-Val-CAC, being far more abundant in cauda sperm than in caput [15–17]. We therefore repeated small RNA-Seq for caput and cauda sperm samples obtained from males three or six weeks after mating. Importantly, we confirmed the same overall differences between these sperm samples in their small RNA payload (**S10 Fig**), demonstrating that at least this observation is not an artifact of some transient early process in virgins. That said, other aspects of the sperm maturation process may differ between virgins and mated males, emphasizing the importance for reproduction studies to explicitly state the mating status of males being used.

Secondly, our work has important technical implications for studies of the sperm methylome, as future efforts focused on cytosine methylation in sperm must contend with the possibility of contamination by cell-free DNA. Although most such studies do not utilize caput epididymal sperm, it is well known that different labs differ in whether “mature sperm” used for molecular studies or IVF are isolated only from the cauda epididymis, or from a mixture of cauda and vas deferens. Given our findings here with vas deferens sperm, it is clear that dissections that capture variable lengths of the vas deferens will result in variability in sperm methylation due to the contamination of these samples by DNA with a somatic cell methylation program. In addition, although we found contaminating DNA specifically in the caput and vas of virgins, it is plausible that cell-free DNA could be more persistent or produced in different parts of the epididymis in other strain backgrounds or in males subject to different diets or stressors. A simple solution to this issue in future studies would be to treat all sperm samples with DNase I prior to genomic DNA isolation for methylation analyses.

## Materials and methods

### Ethics statement

Animal husbandry and experimentation was reviewed, approved, and monitored under the University of Massachusetts Medical School Institutional Animal Care and Use Committee (Protocol ID: A-1833-18).

### Mice

Unless otherwise specified, tissues were obtained from 10–12 week old male FVB/NJ or C57Bl6/J mice.

### Dissection and sperm purification

FVB mice, euthanized at 10–12 weeks of age (unless otherwise noted) according to IACUC protocol, were dissected into four segments that roughly corresponded to caput, corpus, cauda, and vas deferens. Caput, corpus, and cauda epididymis as well as vas deferens were placed into Donners complete media [37] and tissue was cleared of fat and connective tissue before incisions were made using a 26G needle while keeping the bulk tissue intact. Tissue was gently squeezed allowing sperm to escape into solution. After incubation at 37°C for 1 hour, sperm containing media was transferred to a fresh tube and collected by centrifugation at 5000rpm for 5 minutes followed by a 1X PBS wash. To eliminate somatic cell contamination, sperm were subjected to a 1mL 1% Triton X-100 incubation 37°C for 15 mins with 1500 rpm on Thermomixer and collected by centrifugation at 5000rpm for 5 minutes. Somatic cell lysis was followed by a 1x ddH2O wash and 30 second spin 14000 rpm to pellet sperm.

### Isolation of caput sperm using a discontinuous Percoll gradient

Sperm collection from the caput epididymis is performed as described above, but without somatic cell lysis. Instead, caput sperm are purified using a Percoll gradient as described [38] to separate somatic cells away from sperm. Briefly, the caput sperm suspension is carefully layered over a discontinous Percoll gradient containing 45% Percoll (upper phase) and 90% Percoll (lower phase). After centrifugation for 25 min at room temperature (650xg), the interphase containing caput sperm is washed with 1x PBS and prepped for downstream analysis.

### Testicular spermatocyte and spermatid isolation

For each isolation, two testes were acquired from one FVB/NJ mouse at 10–12 weeks of age. Cell suspension was prepared by incubating the testes without their tunica albuginea in 5 ml elutriation buffer (100 mM NaCl, 45 mM KCl, 6 mM Na_2_HPO_4_, 0.6 mM KH_2_PO_4_, 0.23% Sodium DL-Lactate, 0.1% Glucose, 0.1% BSA, 0.011% Sodium Pyruvate, 1.2 mM MgSO_4_ and 1.2 mM CaCl_2_) containing 25 μg/ml liberase (Roche Diagnostics GmbH) for 30 min at 37°C with gentle agitation every 5 min. The cell suspension was mixed by pipetting 20 times with a 10-ml plastic pipette. After homogenization by pipetting 10 times through a P1000 pipette, the single cell suspension was filtered twice through a 40-μm cell strainer (Fisher Scientific) on ice and centrifuged at 1500 rpm at 4°C for 10 min, and then the pellet was resuspended with 20 ml elutriation buffer. Separation of testis cell populations was performed by centrifugal elutriation using a JE-5.0 elutriation system and a 4-ml standard elutriation chamber (Beckman Coulter). The assembly of the system followed the manufacturer’s instruction. The precise elutriation conditions are as follows: Fractions 1–3 were run at 3000 rpm and fractions 4–5 were run at 2000 rpm. The flow rate was 14, 18, 31, 23, and 40 ml/min for fractions 1–5, respectively. During elutriation, the elutriation chamber was maintained at 4°C and the cells were collected into 50-ml conical polypropylene tubes that were packed on ice. Cells in tubes of fractions 3 to 5 were pelleted by centrifugation at 1500 rpm at 4°C for 10 min. All pellets from the same fraction were combined and resuspended in 200 μl of elutriation buffer. Percoll (Sigma-Aldrich) gradient (23–35%) was prepared by using a Gradient Master 108 (Biocomp) following standard program: S1/1, 2:26 (time), 82.0 (angle), 13 (rpm). After loading the cell suspension, centrifugation was carried out with SW40Ti Rotor (Beckman Coulter) at 11,000 rpm at 4°C for 15 min. The cells were then collected in 15-ml conical polypropylene tubes and pelleted by centrifugation at 1500 rpm at 4°C for 15 min. The cell pellets were stored at -80°C and ready for DNA extraction. Based on cell morphology and small RNA data, Fraction 5 was determined to correspond to primary spermatocytes, with fractions 4 and 3 being relatively early and late round spermatids, respectively.

For isolation of the first wave of testicular spermatozoa from postnatal day 35, a cell suspension was prepared by dissecting testes from postnatal day 35 males into 35mm dish containing 150 mM NaCl, removing tunica albuginea, and dissociating tissue using 22G needle and 3 mL syringe with 150 mM NaCl. Cell suspension was then pipetted into a 15 mL conical tube to allow for large tissue to settle. Once settled, top 1mL was loaded onto 52% isotonic Percoll solution. Samples were ultracentrifuged at 15,000 rpm for 10 min at 10 ^o^C. Following ultracentrifugation, pellet was carefully isolated, washed with 150 mM NaCl to remove residual Percoll, resuspended in PBS, and assessed for purity using microscopy (see S1A Fig in [17] for typical preparation) before undergoing the genomic DNA isolation protocol.

### Genomic DNA isolation

750 μL Extraction Buffer (4.24M Guanidine Thiocyanate, 100 mM NaCl, 1% N-Laurylsarcosine, 150 mM freshly prepared DTT, 200 μg/mL Proteinase K) was added to sperm pellets and incubated for 2 hours at 56 ^o^C with shaking. Samples were then allowed to equilibrate to room temperature after which 600 μL isopropanol was added to precipitate DNA followed by a 15 mins spin at 14,000rpm. Supernatant was carefully discarded and pellets were washed 2x with 80% EtOH, dried, and resuspended in 10 mM Tris pH8 (500 μL) with 5 μL RNase A (Qiagen 19101) for 2 hours after which samples were treated with 5 μL Proteinase K (Qiagen 19131) overnight. Following phenol chloroform extraction (UltraPure Phenol:Chloroform:Isoamyl Alcohol ThermoFisher 15593031) using phase lock gel tubes (Quantabio 10847–802), aqueous phase was transferred to a fresh tube containing 1.5 μL glycogen (20 mg/mL), 1.3 μL 5 M NaCl, and 100% EtOH. Genomic DNA integrity was determined using NanoDrop and quantified for downstream applications using Qubit (DNA BR)

### Whole genome bisulfite sequencing

For each population we collected sperm samples from seven different males, isolated genomic DNA, and prepared libraries for whole genome bisulfite sequencing (WGBS); Bisulfite converted DNA was prepared according using the EZ DNA Methylation-Lightning Kit (Zymo D5030). Library construction was performed using Accel-NGS Methyl-Seq DNA Library Kit (Swift) according to the manufacturer’s instructions. 30ng bisulfite converted DNA was used and barcoded following the (Swift) manufacturer’s instructions including 6 rounds of PCR. All final libraries were analyzed by Fragment Analyzer and quantitated using the Qubit as well as the KAPA Library Quantitation qPCR kit.

### Data analysis

Sequences were trimmed with Trim Galore (v0.4.4; Cutadapt v1.9.1)—Swift libraries were trimmed by 10 bp from their 5’ ends for both R1 and R2. The non-CG methylation levels were consistently very low (~0.2%) indicating good bisulfite conversion rates. The resulting trimmed sequences were mapped to the mouse GRCm38 genome using Bismark [39] (v0.17.0); CpG methylation calls were extracted and analysed using SeqMonk (www.bioinformatics.babraham.ac.uk/projects/seqmonk/). See **S1 Table** for summary statistics of read counts, mapping and duplication rates, and bisulfite conversion efficiency. Analyses in **Figs 1**, **S1 and S2** were carried out using evenly-spaced 200 bp tiles, using only tiles with at least 1 CpG and at least 10 methylation-informative reads in every one of the seven datasets. For analyses in **Figs 2B** and **S3**, each CpG island was assigned to the nearest gene based on proximity to the transcription start site (within 50 kb).

### Pyrosequencing

Pyrosequencing was performed using the PyroMark Q24 (Qiagen: 9001514) according to the manufacturer’s instructions. Bisulfite converted DNA was prepared using the EZ DNA Methylation-Lightning Kit (Zymo D5030). PCR was performed with 10–20 ng of bisulfite-converted material for each locus of interest (**S4 Table**). Primers (IDT) were designed using the PyroMark Assay Design software (Qiagen).

### *Ex vivo* sperm incubations

Prior to genomic DNA extraction, sperm were incubated for 4 hours at 37°C in the following conditions: Donners complete media [37], Donners complete media supplemented with either 200 μM SAM, additional sodium pyruvate (10 mM), additional sodium _DL_-Lactate (1% vol/vol), NaHCO_3_ (200mM) as well as Donners complete buffered to pH 6.5, 7, and 7.4. Additional conditions include: Donners basic (stock solution) [37], Donners basic supplemented with NaHCO_3_ (final 25 mM), Donners basic supplemented with BSA (final 20 mg/mL), Donners basic supplemented with Sodium _DL_-Lactate (final 0.53% vol/vol), and finally Donners basic buffered to pH 6.5, 7, and 7.4.

DNase treatment of caput sperm was performed prior to gDNA isolation as per manufacturer’s instructions (Qiagen79254). After DNase treatment, sperm were spun for 30 sec to (14000 rpm), supernatant aspirated, and sperm samples processed for genomic DNA extraction (see: ***Genomic DNA isolation****)*.

### Visualization of cell-free DNA and immunostaining

After sperm prep, samples were dried onto VWR Superfrost Plus Micro Slides (48311–703), fixed for 10 minutes in 4% paraformaldehyde solution, washed 3x with PBS, and permeabilized for 10 min with 0.1% triton in PBS. Blocking was performed with 10% BSA in PBS for 1 hour at room temperature in a humidified chamber. Staining with primary antibody, Anti-Histone H3 (citrulline R2 + R8 + R17 (Abcam ab5103), was performed for 2 hours at room temperature in 1% BSA PBSt at a concentration of 1:250 in a humidified chamber. Primary antibody was decanted and slides washed three times with PBS for 5 minutes each. Goat anti-Rabbit 488 secondary antibody (Invitrogen A11008) was performed for 1 hour at room temperature at a concentration of 1:500 in a humidified chamber. Secondary antibody was decanted and slides washed three times with PBS for 5 minutes each followed by VECTASHIELD Antifade Mounting Medium with DAPI (Vector Labs H-1200), sealing coverslip, and imaging on the Zeiss Axioskop 2 Plus. All studies were repeated with at least two distinct sperm samples. Exposures for different sperm preps were captured using identical light intensity and exposure times, set to enable visualization of cell-free DNA in DAPI images, and set just below saturation for cit-H3 images. Note that secondary alone controls for cit-H3 were clean (not shown).

### Histology

Virgin or retired breeders of approximately the same age were anesthetized and perfused with phosphate-buffered saline (PBS) followed by 4% paraformaldehyde (PFA)/PBS. Epididymides were explanted and further incubated in 4%PFA/PBS at 4°C overnight. After washing the excess of PFA with PBS, the sample was incubated at 4°C in 30% sucrose, 0.002% sodium azide in PBS until organ sank to the bottom of the tube. The sucrose solution was replaced and once organ remained at the bottom of the tube the same volume of optimal cutting temperature compound (OCT) was added to the vial and kept ON at 4C under agitation. Samples were then mounted in OCT and frozen at −80°C until sectioning. Sectioning was done at a thickness of 5 μm by the UMASS morphology core. Slides were stored frozen at -20°C.

Slides were placed at a 37°C warm plate for 10 minutes to ensure proper attachment of the section to the slide, then washed three times for 5 minutes in PBS 0.02% tween 20 (PBS-T) to remove OCT, followed by DAPI stain (3 μM 4′,6-diamidino-2-phenylindole in PBS) for 5 min. After washing off the excess of DAPI with PBS-T, slides were mounted with ProLong gold Antifade (Thermofisher P36930) and imaged the following day. **S8 Fig** shows characteristic images for a range of exposure times to illustrate the presence of a DAPI rim in virgin caput samples, but not mated caput or cauda epididymis and show that this rim is not simply an artifact of different exposure times used for different samples.

### Small RNA sequencing

Males from the same litter were split into the following groups: not mated, mated and recovered for three weeks, and mated and recovered for six weeks. Mating was confirmed by the formation of blastocysts in culture. All males were dissected at the same age (14 weeks). Caput and cauda sperm were isolated as described and small RNA sequencing was performed. Isolation of 18–40 nts small RNAs was carried out as previously described [17]. Size selection by purification of RNAs from 15% polyacrylamide-7M urea denaturing gels and sequencing library preparation using Illumina’s TruSeq Small RNA Library Preparation Kit.

## Supporting information

S1 Table WGBS read depth and bisulfite conversion efficiencies. Deep sequencing statistics for WGBS datasets used in this manuscript.
(XLSX)

S2 Table Location of caput sperm DMRs. Rows show genomic coordinates, nearest genes (within 50 kb), and methylation levels in all seven sperm populations for all 200 bp tiles that are at least 20% hypo- or hyper-methylated in caput sperm compared to cauda sperm.
(XLSX)

S3 Table Methylation levels at CpG islands. Rows show methylation levels and location of nearest gene for annotated CpG islands.
(XLSX)

S4 Table Oligonucleotides used for pyrosequencing. Sequences for pyrosequencing primers used in **Figs 3 and 4**.
(XLSX)

S1 Fig Cauda sperm methylome is concordant with published sperm methylation data. Scatterplot shows methylation levels for 200 bp windows tiled across chromosome 1, comparing data for cauda sperm from Wang *et al* [20] (x axis) with data from this study. Our data recapitulate prior findings, with a good overall correlation of ~0.95. Moreover, although meaningful methylation differences might be expected given the differing ages and strain backgrounds in the two studies, inspection of windows with methylation differences reveals that all loci examined exhibit substantial differences in sequencing coverage between the two datasets, arguing that most such tiles in this comparison reflect unreliable measurement in one or the other dataset. The general agreement between our data and prior genome-wide datasets, along with our recovery of known features of the sperm methylome (global methylation punctuated by unmethylated CpG islands), further emphasize the quality of our dataset.
(TIF)

S2 Fig Correlations between all seven germ cell methylation datasets. Scatterplots are shown as in **Fig 1D and 1E**, for all pairwise comparisons in this dataset.
(TIF)

S3 Fig GO enrichments for genes associated with aberrant CpG island methylation in caput sperm. For all CpG islands exhibiting >20% methylation differences between caput and cauda sperm, nearest genes were identified and enriched gene ontology categories were identified using Funcassociate [40]. Bar plots show p values (expressed as -log_10_) for selected categories enriched among hyper (red) and hypo (green) methylated CpG islands.
(TIF)

S4 Fig Genomic context for imprinted loci and loci targeted for followup. A) WGBS data for genomic loci selected for targeted follow-up. At each locus, red and blue dashes represent individual reads for a given CpG, with red and blue showing methylated or unmethylated reads respectively. Top panels show a wider view of the genomic context, with boxes indicating regions shown in the zoom-in bottom panels. For these five regions, methylation levels obtained from the WGBS dataset are shown for caput and cauda sperm samples, along with methylation levels obtained in followup pyrosequencing validation (using the average of all replicates shown in **Fig 3A**). For all five loci, pyrosequencing qualitatively confirmed the methylation trends observed in WGBS; while there was some quantitative disagreement (eg 83% vs 42% for methylation at *Tdrd12* in caput sperm), we note that values inferred for small numbers (~4–5) of CpGs in the WGBS dataset are expected to be somewhat noisy given the relatively low sequencing depth. B) As in panel (A), but for selected imprinted genes. Notably, while testicular germ cells and corpus and cauda sperm exhibit the expected 0% or 100% methylation, our caput sperm (and to a lesser extent vas deferens sperm) data are closer to the 50% methylation expected of somatic cells. C) Scatterplot comparing methylation levels at imprinting control regions (using [41] for ICR coordinates) in primary spermatocytes (x axis) and caput sperm (y axis). Caput sperm exhibit a clear shift from the expected sperm profile (with either ~0% or ~100% methylation) towards the ~50% methylation characteristic of somatic cells. D) Loci studied in Ariel *et al*, 1994. As our study was initially motivated by the findings of cytosine methylation changes occurring during post-testicular sperm maturation as reported by Ariel *et al*, we show WGBS data for the two loci documented in detail in that study. However, the assay used by Ariel *et al*–digestion using a methylation-sensitive restriction enzyme following by PCR across the cut site–reports on a single CpG, and our data are not deeply-sequenced enough to confidently assess methylation levels at the two individual CpGs in question. That said, pyrosequencing at one locus–*Pgk2* –did not recapitulate the findings reported in Ariel *et al*: we found no difference in methylation of this one CpG in caput vs cauda sperm (31% vs 27%), while Ariel *et al* reported minimal methylation at this site in caput sperm along with higher methylation in cauda sperm. There are countless potential technical differences that might account for this, including strain differences (CD1 vs FVB and C57), mating status and age (not specified in Ariel *et al*), sperm preparation (Ariel *et al* used sonication to deplete somatic cells, we used both detergent and Percoll in different preparations), or genomic DNA extraction (we used 150 mM DTT for sperm lysis, Ariel *et al* used significantly lower reducing agent– 0.001% v/v β-ME), and of course the methylation readout (quantitative pyrosequencing vs qualitative PCR following methylation-sensitive restriction digestion).
(TIF)

S5 Fig The caput methylation profile is stable for at least ten months in unmated animals. Pyrosequencing data for the two indicated target loci in four unmated males at ten months of age, confirming that the caput methylome is stable throughout a typical male’s lifespan.
(TIF)

S6 Fig Caput sperm methylation status is stable under many different buffer conditions. Pyrosequencing data for caput and cauda sperm samples. Caput sperm samples were either processed for genomic DNA shortly after isolation, or were incubated for four hours at 37°C in various buffer conditions, as indicated. Buffer conditions were based on either Donners Basic (DB) or Donners Complete (DC), and were supplemented with various levels of NaHCO_3_, sodium pyruvate, sodium DL-Lactate, or adjusted to pH 6.5, 7.0, or 7.4 (red triangles). Although not indicated in the figure, cauda sperm samples (right) included samples subject to most of the buffer incubations used for caput sperm samples, none of which affected methylation in these samples.
(TIF)

S7 Fig Cell-free DNA contaminates caput but not cauda sperm preps. For all samples, left panels show DIC images and right panel shows DAPI staining. Panels show mock-treated and DNase-treated caput sperm (A) and cauda sperm (B), revealing extensive DNase-sensitive background “fog” and fibrous tangles in the mock-treated caput sperm (see also **Fig 4A**) but not cauda sperm. In the case of cauda sperm, DAPI staining was completely confined to sperm heads, and no webs of DNA were observed even at the highest exposures. Also notable here is the high purity of our sperm samples, with no round DAPI-stained nuclei contaminating the field of sperm heads in any of the four samples. These representative samples illustrate the >99% purity of our sperm preparations that we routinely ensure by microscopic examination prior to processing for molecular studies.
(TIF)

S8 Fig In situ imaging of DAPI staining in the caput and cauda epididymis. A) Images of DAPI-stained epididymis sections from the indicated samples: virgin caput epididymis (3 samples shown), mated caput, virgin cauda, and mated cauda. Top five rows are images taken from C57Bl6/J animals (at 25 weeks of age), where the “rim” of DAPI in the caput epididymis is more prominent, while bottom row shows a typical FVB caput epididymis (10 weeks of age) with a noticeable but dimmer DAPI rim. In each row, five exposures are shown from lowest (left) to highest (right) exposure to show that the virgin caput DAPI rim is not an artifact of mismatched exposures. B) Higher-resolution images are reproduced from the highest exposures from panel (A), as indicated. These images emphasize the presence of a DAPI-stained rim (examples highlighted with yellow brackets) in virgin but not mated caput epididymis. Although the presence of the DAPI rim corresponds to anatomical regions where sperm preparations are contaminated by cell-free DNA–virgin caput epididymis and vas deferens, but not cauda or mated caput–the physical nature of this rim is unclear. We note the presence of occasional nuclei within this rim in locations presumably corresponding to apically-located clear cell nuclei, indicating that the DAPI rim largely colocalizes with the cell bodies of the caput epididymal epithelium. Yet the absence of this DAPI rim in the caput epididymis of mated animals shows that this staining is not nonspecific background staining or autofluorescence. Further defining the nature and subcellular localization of this material will likely require super-resolution imaging and additional DNA staining approaches.
(TIF)

S9 Fig Resolution of the caput methylome by DNase I treatment. A) Pyrosequencing data show methylation at the three indicated target loci in testicular spermatozoa, caput, corpus, and cauda epididymal sperm, and vas deferens sperm, as indicated. All samples were obtained from 10–14 week old FVB males, and sperm were DNase-treated prior to genomic DNA isolation. In all cases DNase treatment completely eliminated methylation differences between the various samples. See also **Fig 4C**. B) Data for the indicated samples obtained from 10–14 week old C57 males. Data here are shown for samples either mock-treated (left samples) or DNase-treated (right samples, as indicated) prior to genomic DNA extraction. See also **Fig 4D**.
(TIF)

S10 Fig Mating does not impact the small RNA payload of caput sperm. Several previous studies have documented significant differences between the RNA payload of caput and cauda sperm, including dramatically lower levels of genomically-clustered microRNAs in caput sperm relative to both testicular and cauda sperm [15–17]. To determine whether this unusual small RNA profile is unique to caput sperm obtained from virgins, we obtained caput and cauda epididymal sperm from virgin males as well as males three and six weeks after successful mating, and characterized small RNAs by deep sequencing. A-B) Scatterplot compares small RNA levels in virgin caput sperm to levels in caput sperm 3 (A) or 6 (B) weeks after mating. Data are shown for all small RNAs with an abundance of at least 10 ppm in virgin caput sperm. C) Scatterplot shows enrichment/depletion in cauda sperm vs. caput sperm (calculated as log_2_(Cauda+1)/(Caput+1)) for virgin males (x axis) compared to males 6 weeks after mating (y axis). Overall enrichments for small RNAs in cauda or caput sperm remain highly correlated after mating. D) Example of cauda-enriched microRNAs from the X-linked miR-465 and miR-880 clusters. Enrichment in cauda sperm, and absence from caput sperm, was unaffected by mating status.
(TIF)
