# Supplementary figures and images for: Stability of the cytosine methylome during post-testicular sperm maturation in mouse
Source: PLoS Genet. 2021 Mar 4;17(3):e1009416. doi: 10.1371/journal.pgen.1009416 (PMC7963034; doi:10.1371/journal.pgen.1009416)

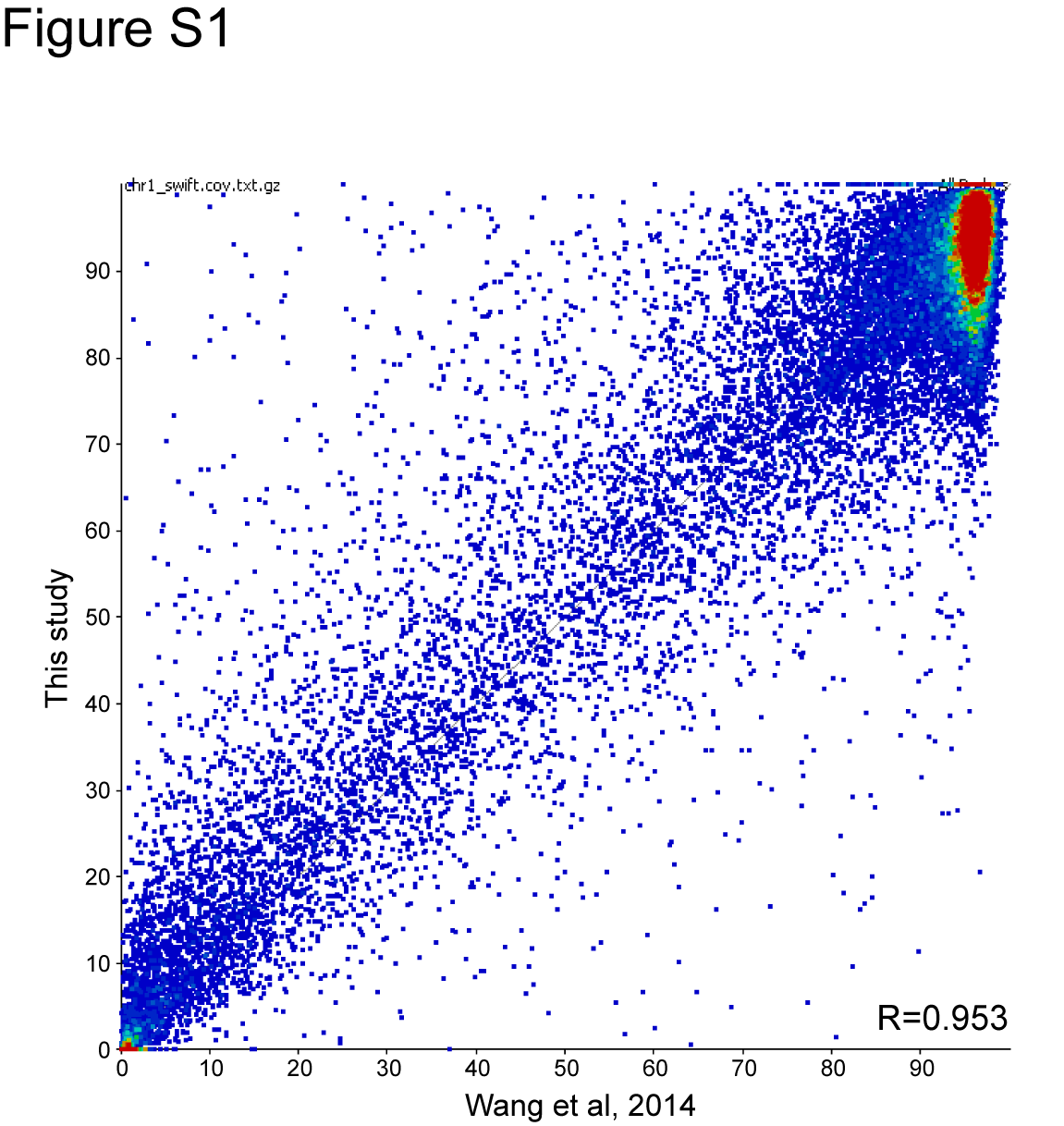

Supplement: S1 Fig — Scatterplot shows methylation levels for 200 bp windows tiled across chromosome 1, comparing data for cauda sperm from Wang et al [20] (x axis) with data from this study. Our data recapitulate prior findings, with a good overall correlation of ~0.95. Moreover, although meaningful methylation differences might be expected given the differing ages and strain backgrounds in the two studies, inspection of windows with methylation differences reveals that all loci examined exhibit substantial differences in sequencing coverage between the two datasets, arguing that most such tiles in this comparison reflect unreliable measurement in one or the other dataset. The general agreement between our data and prior genome-wide datasets, along with our recovery of known features of the sperm methylome (global methylation punctuated by unmethylated CpG islands), further emphasize the quality of our dataset. (TIF) [file pgen.1009416.s005.tif]

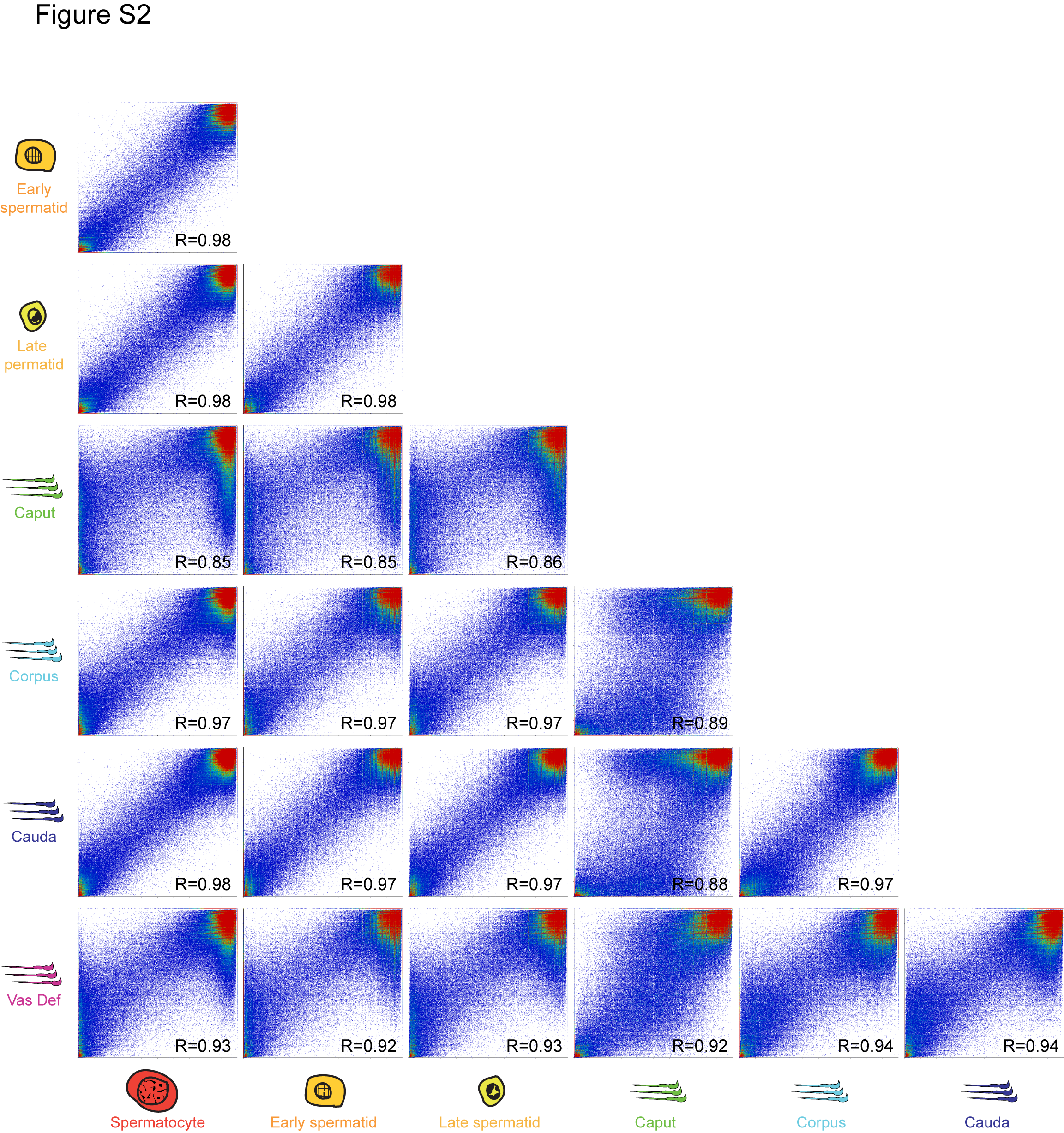

Supplement: S2 Fig — Scatterplots are shown as in Fig 1D and 1E, for all pairwise comparisons in this dataset. (TIF) [file pgen.1009416.s006.tif]

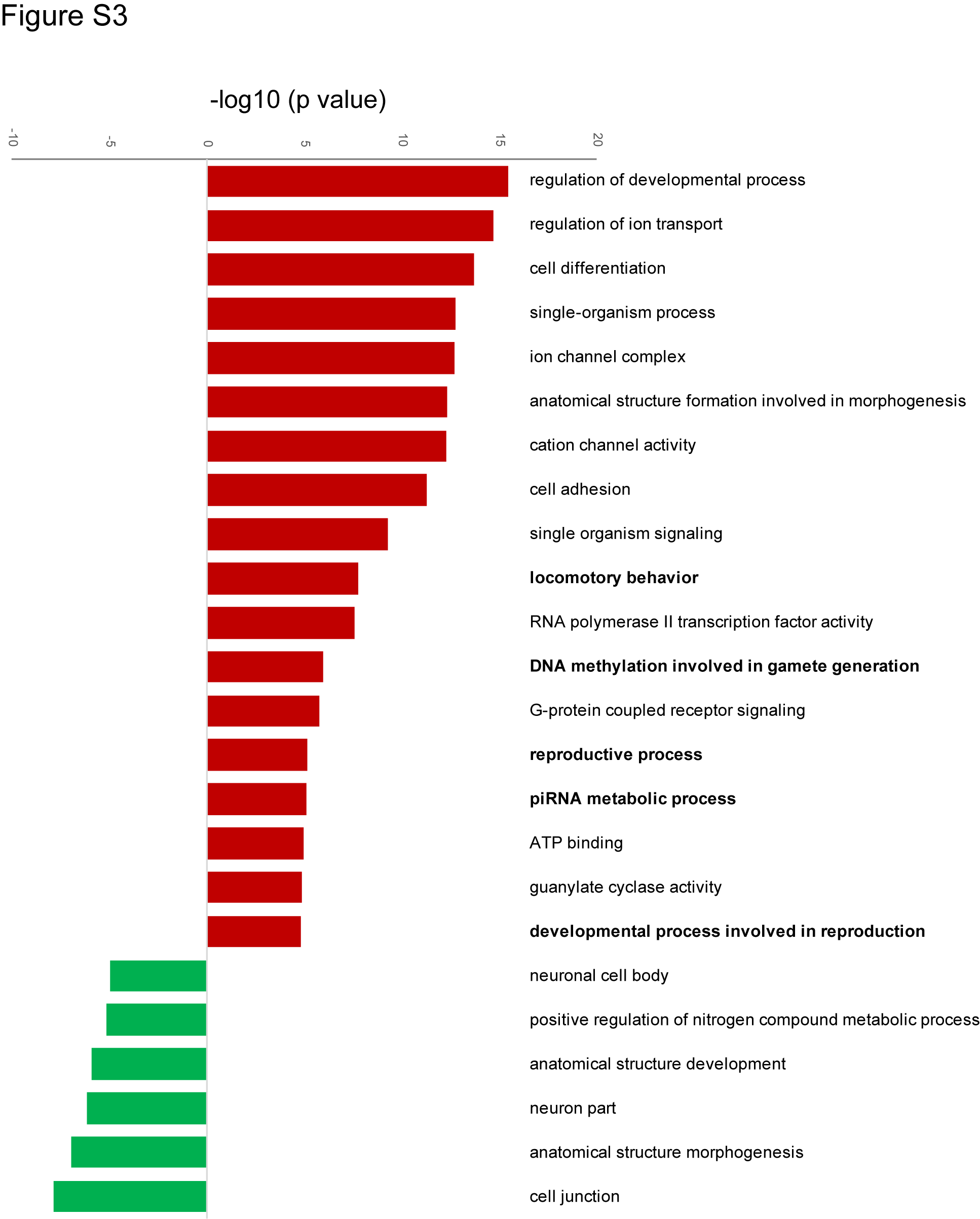

Supplement: S3 Fig — For all CpG islands exhibiting >20% methylation differences between caput and cauda sperm, nearest genes were identified and enriched gene ontology categories were identified using Funcassociate [40]. Bar plots show p values (expressed as -log10) for selected categories enriched among hyper (red) and hypo (green) methylated CpG islands. (TIF) [file pgen.1009416.s007.tif]

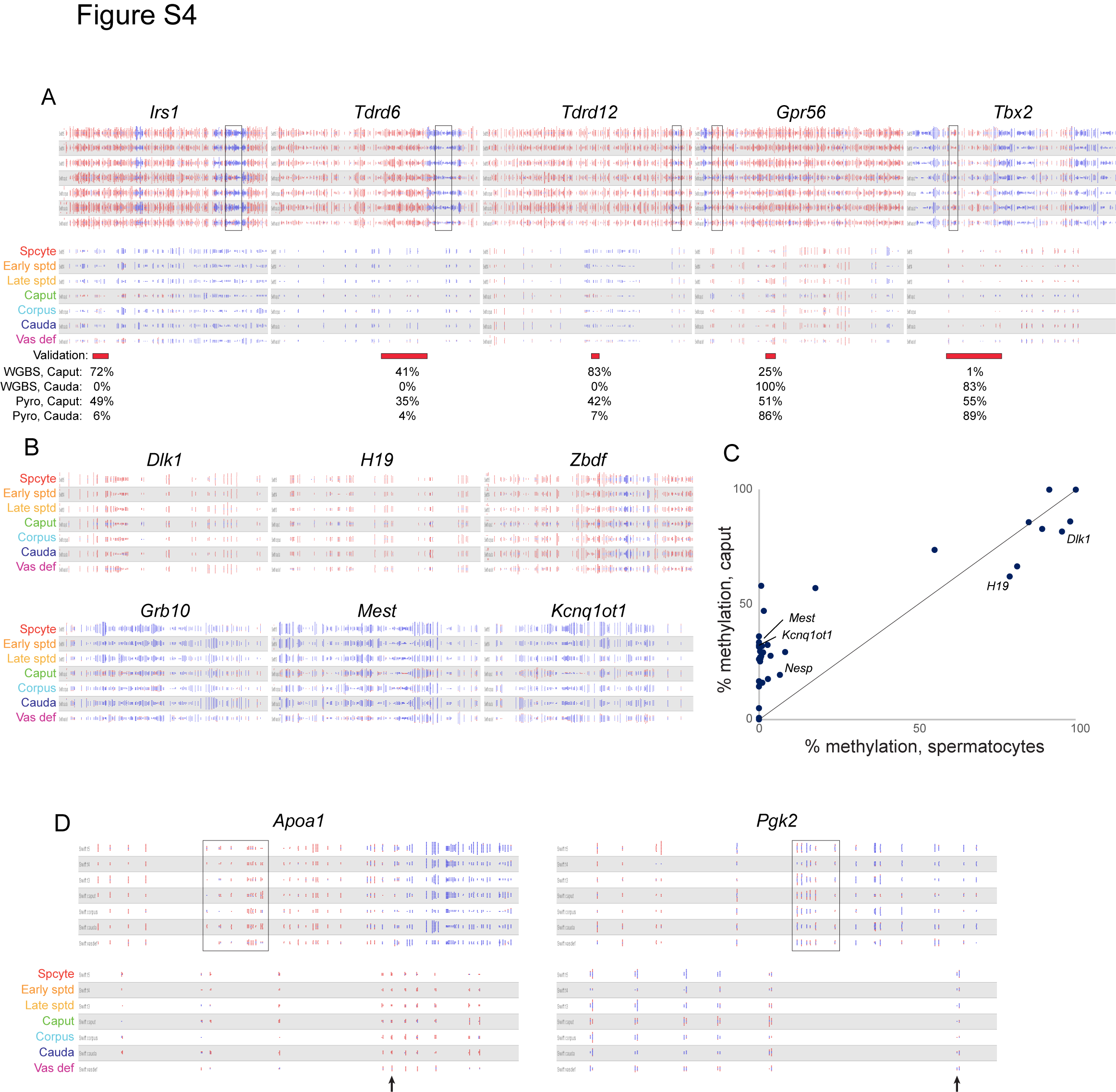

Supplement: S4 Fig — A) WGBS data for genomic loci selected for targeted follow-up. At each locus, red and blue dashes represent individual reads for a given CpG, with red and blue showing methylated or unmethylated reads respectively. Top panels show a wider view of the genomic context, with boxes indicating regions shown in the zoom-in bottom panels. For these five regions, methylation levels obtained from the WGBS dataset are shown for caput and cauda sperm samples, along with methylation levels obtained in followup pyrosequencing validation (using the average of all replicates shown in Fig 3A). For all five loci, pyrosequencing qualitatively confirmed the methylation trends observed in WGBS; while there was some quantitative disagreement (eg 83% vs 42% for methylation at Tdrd12 in caput sperm), we note that values inferred for small numbers (~4–5) of CpGs in the WGBS dataset are expected to be somewhat noisy given the relatively low sequencing depth. B) As in panel (A), but for selected imprinted genes. Notably, while testicular germ cells and corpus and cauda sperm exhibit the expected 0% or 100% methylation, our caput sperm (and to a lesser extent vas deferens sperm) data are closer to the 50% methylation expected of somatic cells. C) Scatterplot comparing methylation levels at imprinting control regions (using [41] for ICR coordinates) in primary spermatocytes (x axis) and caput sperm (y axis). Caput sperm exhibit a clear shift from the expected sperm profile (with either ~0% or ~100% methylation) towards the ~50% methylation characteristic of somatic cells. D) Loci studied in Ariel et al, 1994. As our study was initially motivated by the findings of cytosine methylation changes occurring during post-testicular sperm maturation as reported by Ariel et al, we show WGBS data for the two loci documented in detail in that study. However, the assay used by Ariel et al–digestion using a methylation-sensitive restriction enzyme following by PCR across the cut site–reports on a single Cp [file pgen.1009416.s008.tif]

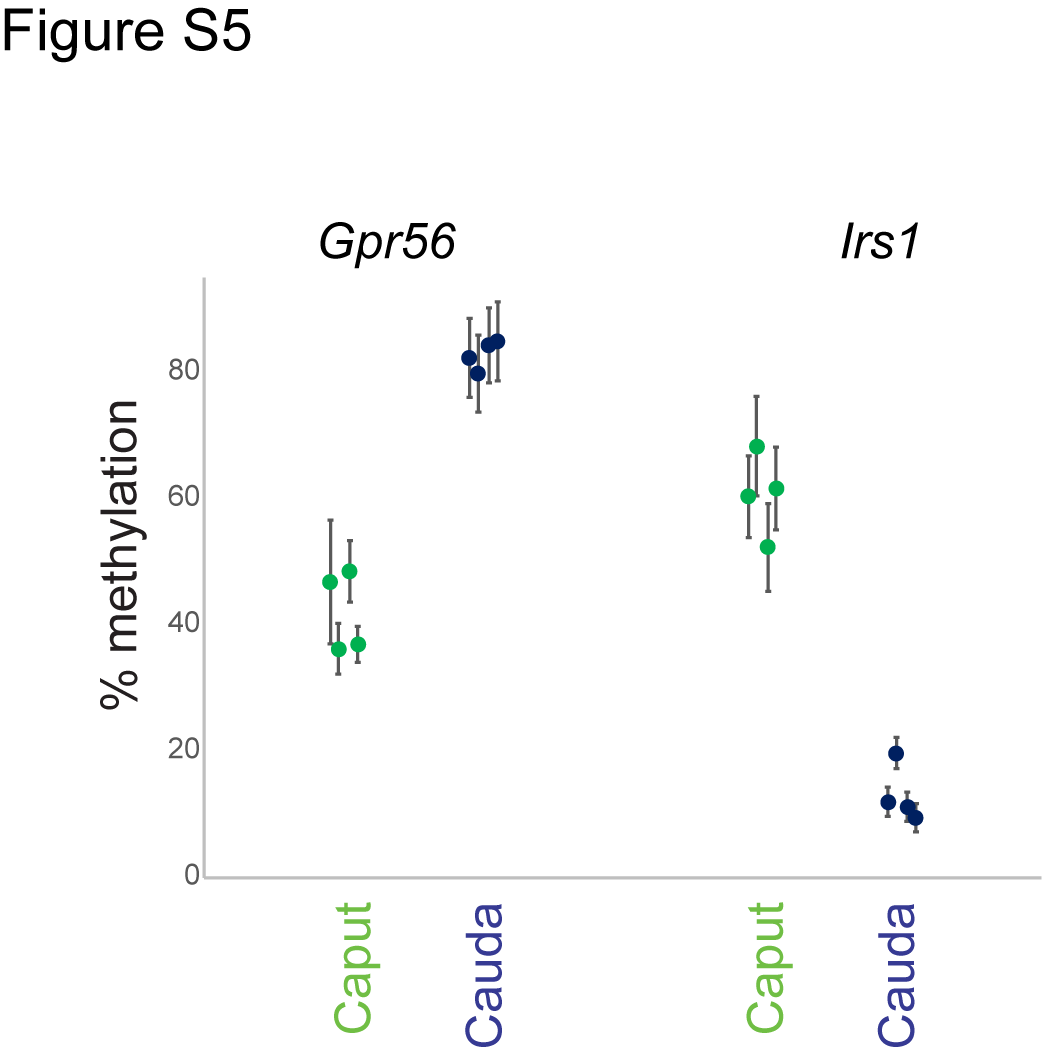

Supplement: S5 Fig — Pyrosequencing data for the two indicated target loci in four unmated males at ten months of age, confirming that the caput methylome is stable throughout a typical male’s lifespan. (TIF) [file pgen.1009416.s009.tif]

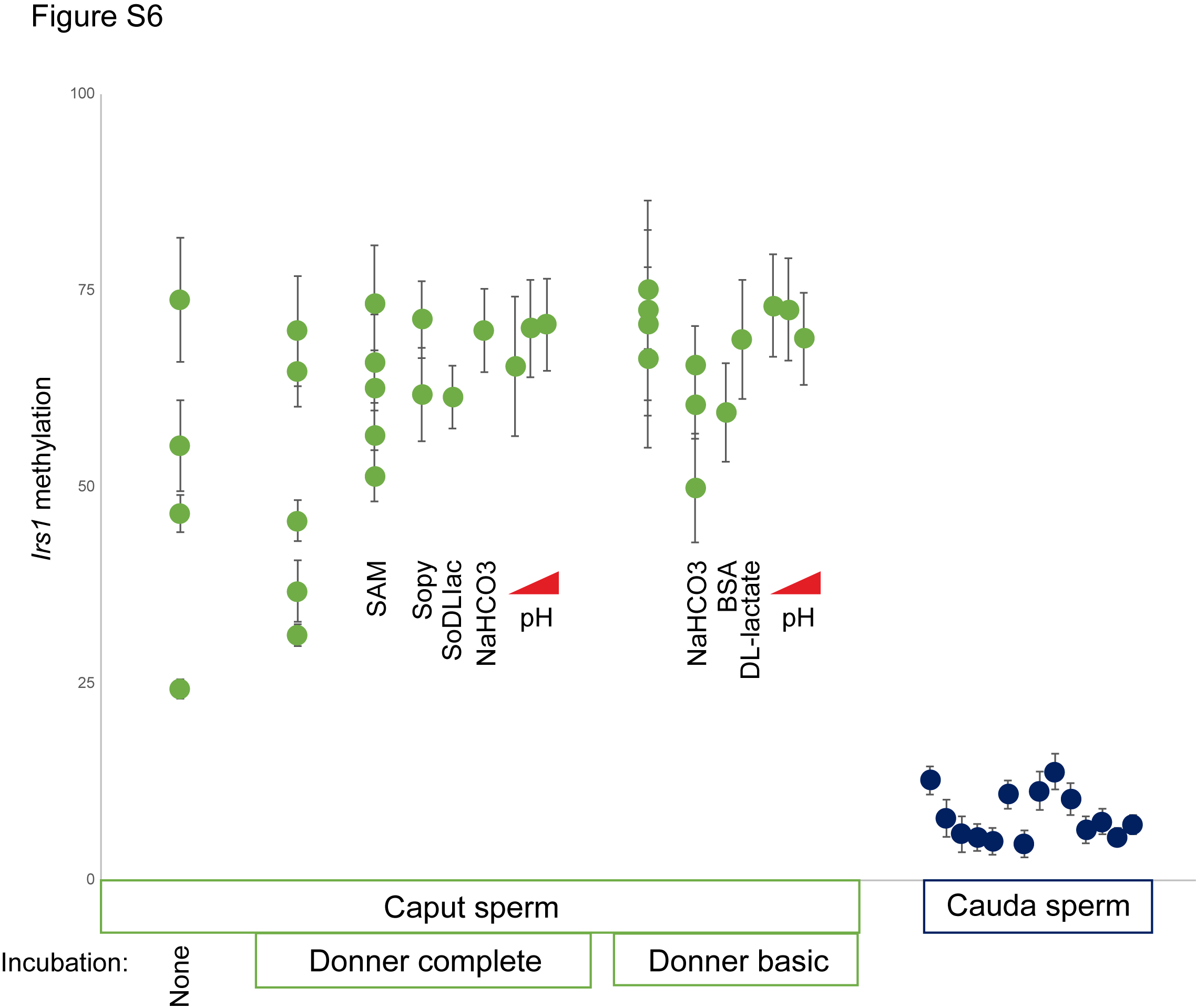

Supplement: S6 Fig — Pyrosequencing data for caput and cauda sperm samples. Caput sperm samples were either processed for genomic DNA shortly after isolation, or were incubated for four hours at 37°C in various buffer conditions, as indicated. Buffer conditions were based on either Donners Basic (DB) or Donners Complete (DC), and were supplemented with various levels of NaHCO3, sodium pyruvate, sodium DL-Lactate, or adjusted to pH 6.5, 7.0, or 7.4 (red triangles). Although not indicated in the figure, cauda sperm samples (right) included samples subject to most of the buffer incubations used for caput sperm samples, none of which affected methylation in these samples. (TIF) [file pgen.1009416.s010.tif]

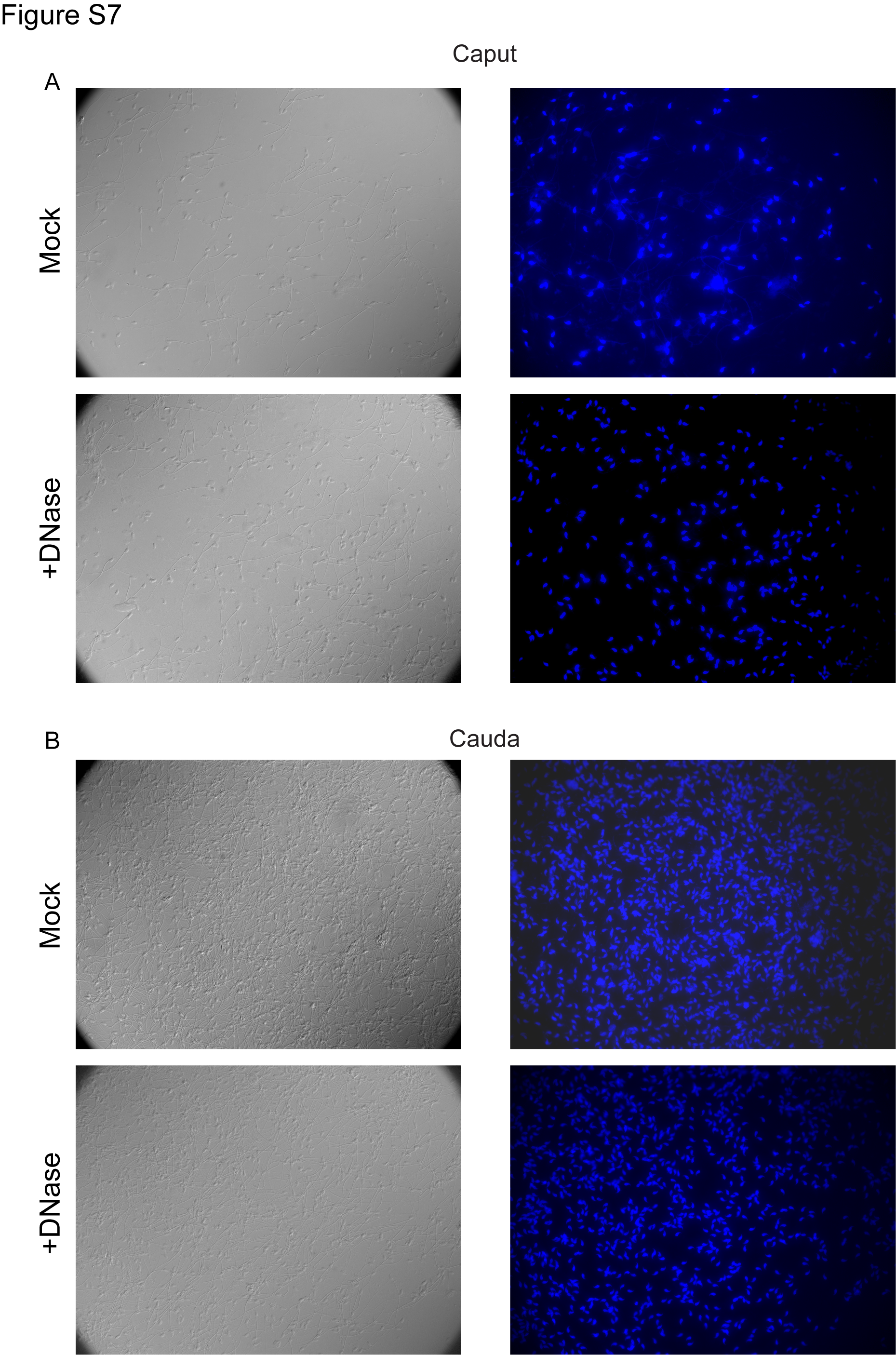

Supplement: S7 Fig — For all samples, left panels show DIC images and right panel shows DAPI staining. Panels show mock-treated and DNase-treated caput sperm (A) and cauda sperm (B), revealing extensive DNase-sensitive background “fog” and fibrous tangles in the mock-treated caput sperm (see also Fig 4A) but not cauda sperm. In the case of cauda sperm, DAPI staining was completely confined to sperm heads, and no webs of DNA were observed even at the highest exposures. Also notable here is the high purity of our sperm samples, with no round DAPI-stained nuclei contaminating the field of sperm heads in any of the four samples. These representative samples illustrate the >99% purity of our sperm preparations that we routinely ensure by microscopic examination prior to processing for molecular studies. (TIF) [file pgen.1009416.s011.tif]

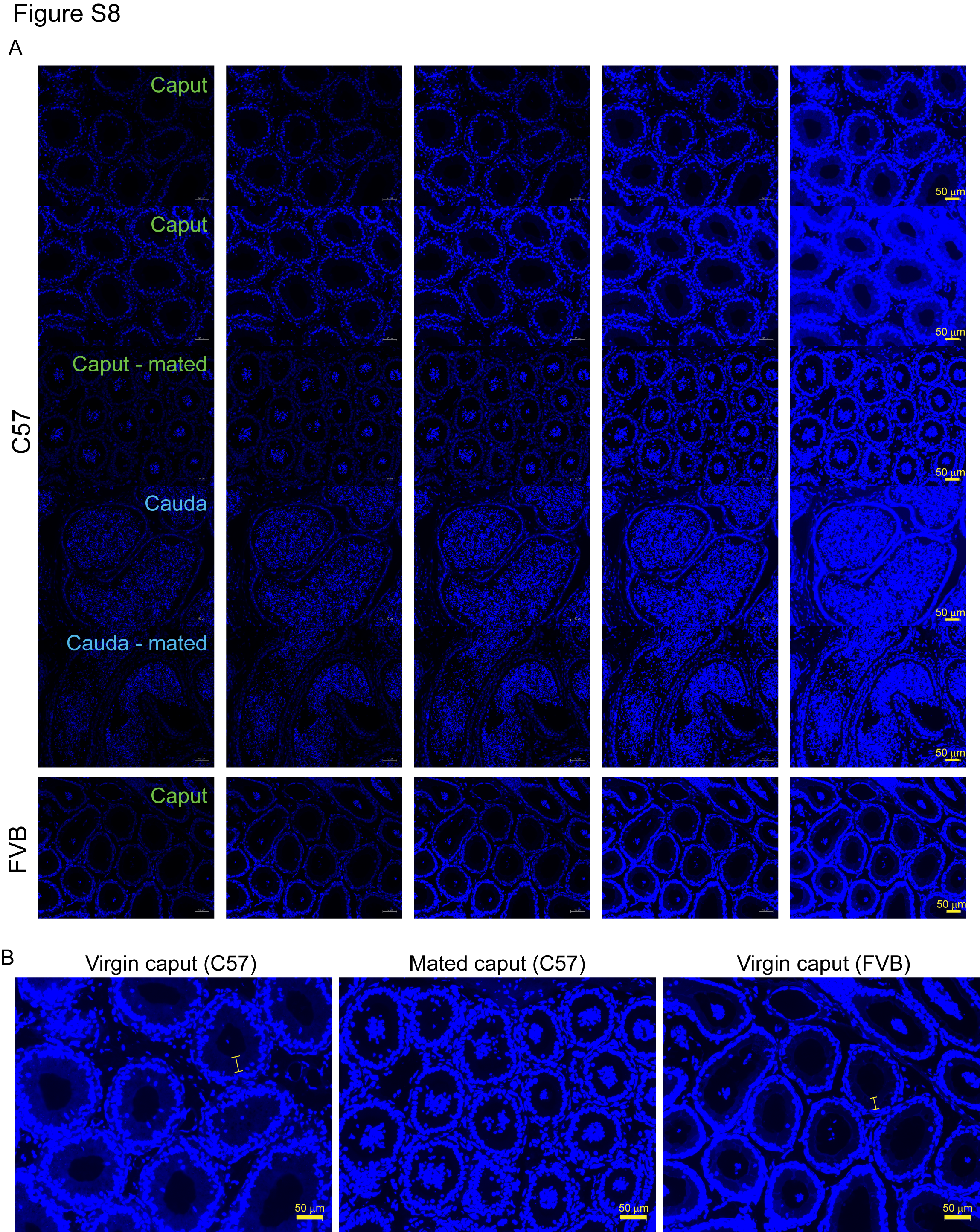

Supplement: S8 Fig — A) Images of DAPI-stained epididymis sections from the indicated samples: virgin caput epididymis (3 samples shown), mated caput, virgin cauda, and mated cauda. Top five rows are images taken from C57Bl6/J animals (at 25 weeks of age), where the “rim” of DAPI in the caput epididymis is more prominent, while bottom row shows a typical FVB caput epididymis (10 weeks of age) with a noticeable but dimmer DAPI rim. In each row, five exposures are shown from lowest (left) to highest (right) exposure to show that the virgin caput DAPI rim is not an artifact of mismatched exposures. B) Higher-resolution images are reproduced from the highest exposures from panel (A), as indicated. These images emphasize the presence of a DAPI-stained rim (examples highlighted with yellow brackets) in virgin but not mated caput epididymis. Although the presence of the DAPI rim corresponds to anatomical regions where sperm preparations are contaminated by cell-free DNA–virgin caput epididymis and vas deferens, but not cauda or mated caput–the physical nature of this rim is unclear. We note the presence of occasional nuclei within this rim in locations presumably corresponding to apically-located clear cell nuclei, indicating that the DAPI rim largely colocalizes with the cell bodies of the caput epididymal epithelium. Yet the absence of this DAPI rim in the caput epididymis of mated animals shows that this staining is not nonspecific background staining or autofluorescence. Further defining the nature and subcellular localization of this material will likely require super-resolution imaging and additional DNA staining approaches. (TIF) [file pgen.1009416.s012.tif]

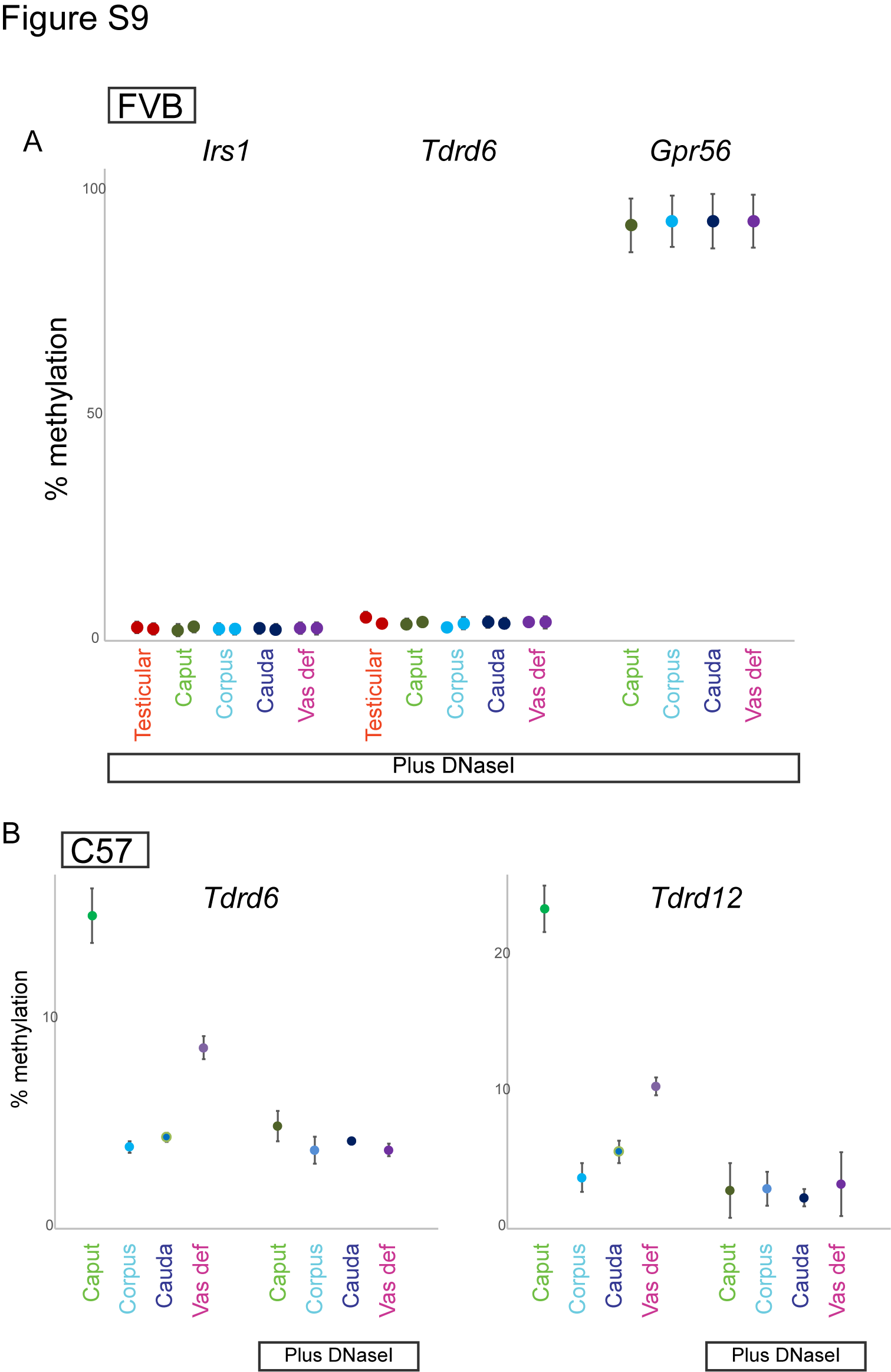

Supplement: S9 Fig — A) Pyrosequencing data show methylation at the three indicated target loci in testicular spermatozoa, caput, corpus, and cauda epididymal sperm, and vas deferens sperm, as indicated. All samples were obtained from 10–14 week old FVB males, and sperm were DNase-treated prior to genomic DNA isolation. In all cases DNase treatment completely eliminated methylation differences between the various samples. See also Fig 4C. B) Data for the indicated samples obtained from 10–14 week old C57 males. Data here are shown for samples either mock-treated (left samples) or DNase-treated (right samples, as indicated) prior to genomic DNA extraction. See also Fig 4D. (TIF) [file pgen.1009416.s013.tif]

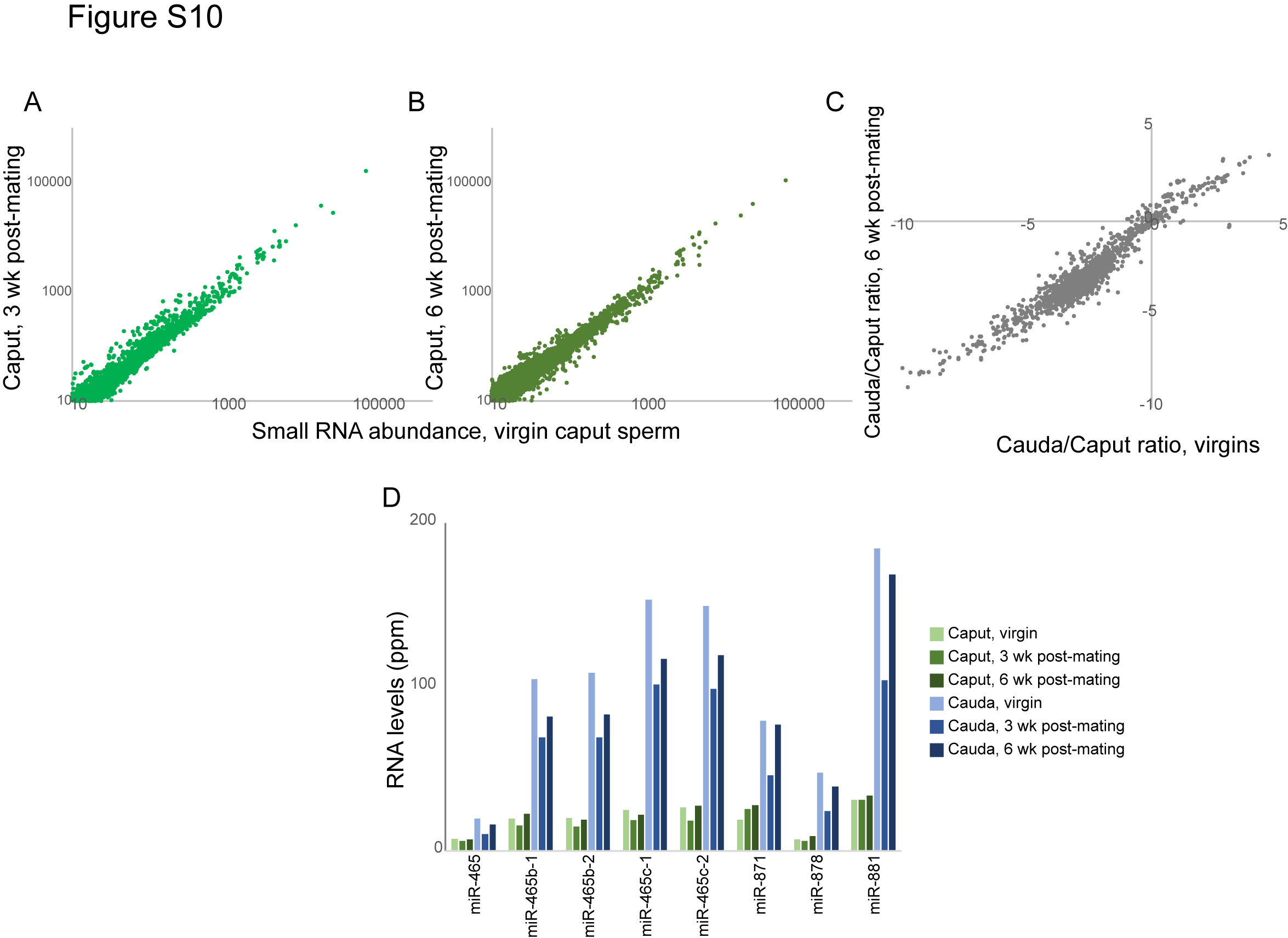

Supplement: S10 Fig — Several previous studies have documented significant differences between the RNA payload of caput and cauda sperm, including dramatically lower levels of genomically-clustered microRNAs in caput sperm relative to both testicular and cauda sperm [15–17]. To determine whether this unusual small RNA profile is unique to caput sperm obtained from virgins, we obtained caput and cauda epididymal sperm from virgin males as well as males three and six weeks after successful mating, and characterized small RNAs by deep sequencing. A-B) Scatterplot compares small RNA levels in virgin caput sperm to levels in caput sperm 3 (A) or 6 (B) weeks after mating. Data are shown for all small RNAs with an abundance of at least 10 ppm in virgin caput sperm. C) Scatterplot shows enrichment/depletion in cauda sperm vs. caput sperm (calculated as log2(Cauda+1)/(Caput+1)) for virgin males (x axis) compared to males 6 weeks after mating (y axis). Overall enrichments for small RNAs in cauda or caput sperm remain highly correlated after mating. D) Example of cauda-enriched microRNAs from the X-linked miR-465 and miR-880 clusters. Enrichment in cauda sperm, and absence from caput sperm, was unaffected by mating status. (TIF) [file pgen.1009416.s014.tif]
